# Characterization of tumour microenvironment reprogramming reveals invasion in epithelial ovarian carcinoma

**DOI:** 10.1186/s13048-023-01270-7

**Published:** 2023-10-10

**Authors:** Yuanfu Zhang, Shu Sun, Yue Qi, Yifan Dai, Yangyang Hao, Mengyu Xin, Rongji Xu, Hongyan Chen, Xiaoting Wu, Qian Liu, Congcong Kong, Guangmei Zhang, Peng Wang, Qiuyan Guo

**Affiliations:** 1 Department of Gynecology, the First Affiliated Hospital of Harbin Medical University, Harbin, 150081 China; 2 College of Bioinformatics Science and Technology, Harbin Medical University, Harbin, 150081 China; 3 Department Gynecology and Obstetrics, the Second Affiliated Hospital of Harbin Medical University, Harbin, 150086 China

**Keywords:** Epithelial ovarian carcinoma, Tissue invasion, Single-cell RNA sequencing, Tumour microenvironment reprogramming, Intratumor heterogeneity

## Abstract

**Background:**

Patients with epithelial ovarian carcinoma (EOC) are usually diagnosed at an advanced stage with tumour cell invasion. However, identifying the underlying molecular mechanisms and biomarkers of EOC proliferation and invasion remains challenging.

**Results:**

Herein, we explored the relationship between tumour microenvironment (TME) reprogramming and tissue invasion based on single-cell RNA sequencing (scRNA-seq) datasets. Interestingly, hypoxia, oxidative phosphorylation (OXPHOS) and glycolysis, which have biologically active trajectories during epithelial mesenchymal transition (EMT), were positively correlated. Moreover, energy metabolism and anti-apoptotic activity were found to be critical contributors to intratumor heterogeneity. In addition, HMGA1, EGR1 and RUNX1 were found to be critical drivers of the EMT process in EOC. Experimental validation revealed that suppressing EGR1 expression inhibited tumour cell invasion, significantly upregulated the expression of E-cadherin and decreased the expression of N-cadherin. In cell components analysis, cancer-associated fibroblasts (CAFs) were found to significantly contribute to immune infiltration and tumour invasion, and the accumulation of CAFs was associated with poorer patient survival.

**Conclusion:**

We revealed the molecular mechanism and biomarkers of tumour invasion and TME reprogramming in EOC, which provides effective targets for the suppression of tumour invasion.

**Supplementary Information:**

The online version contains supplementary material available at 10.1186/s13048-023-01270-7.

## Background

Ovarian cancer is mainly divided into epithelial ovarian cancer (EOC; more than 90%), ovarian sex cord stromal tumour (~ 5%) and ovarian germ cell tumour (~ 3%) according to the pathological phenotype. The EOC patients with conventional surgery have the worst treatment outcome and prognosis compared to ovarian sex cord stromal tumour [1] and ovarian germ cell tumour [2]. Patients are usually at advanced stage when they are diagnosed, which means that EOC has spread to the abdominal cavity and upper abdominal organs, and this is one of the main factors contributing to high mortality [3]. Over nearly 10 years, there have been advances in EOC treatments, and more targeted therapies are on the horizon. For example, poly ADP-ribose polymerase (PARP) inhibitors [4, 5], immunotherapy and heated intraperitoneal chemotherapy (HIPEC) have the potential to improve the survival rate of EOC patients [6, 7]. Therefore, early diagnosis strategies for EOC and the treatment of tumour invasion are important for improving survival. However, the understanding of the mechanisms of tumour cell invasion in EOC patients is limited.

Metabolic reprogramming and tumour tissue invasion are two important hallmarks of cancer [8]. With the progression and invasion of cancer, there are concomitant changes in tissue metabolism. In the tumour microenvironment (TME), cells in different states have specific metabolic requirements [9]. Therefore, there is a specific relationship between intratumor heterogeneity and metabolic reprogramming. However, bulk sequencing reflecting the average expression level of genes in bulky tumours is not sufficient to reflect the specific characteristics of each cell or cell subset in the TME. The application of single-cell RNA sequencing (scRNA-seq) technology in EOC reveals a map of the TME and cellular characteristics [10–12]. Although these studies represent cutting-edge progress in scRNA-seq of EOC, our understanding of TME reprogramming and tissue invasion in EOC still needs to be continually improved.

Malignant cells together with other stromal cells, such as cancer-associated fibroblasts (CAFs), macrophages, and immune cells, constitute the TME. Together, these cells and their complex environment determine tumour progression. For example, mesenchymal cells and CAFs release signalling factors to assist in the invasion and proliferation of tumour cells [13, 14]. Endothelial cells provide blood vessels that can provide nutritional support for cancer cells in a hostile environment. These findings suggest that cell‒cell communication plays an important role in tumour progression and invasion.

To determine the TME reprogramming and invasion mechanisms of EOC, we reanalysed scRNA-seq datasets including two sample cohorts and identified several biomarkers and TME reprogramming characteristics of tumour invasion.

## Results

### Single-cell landscapes demonstrate tumour heterogeneity related to CNVs

**Fig. 1:**
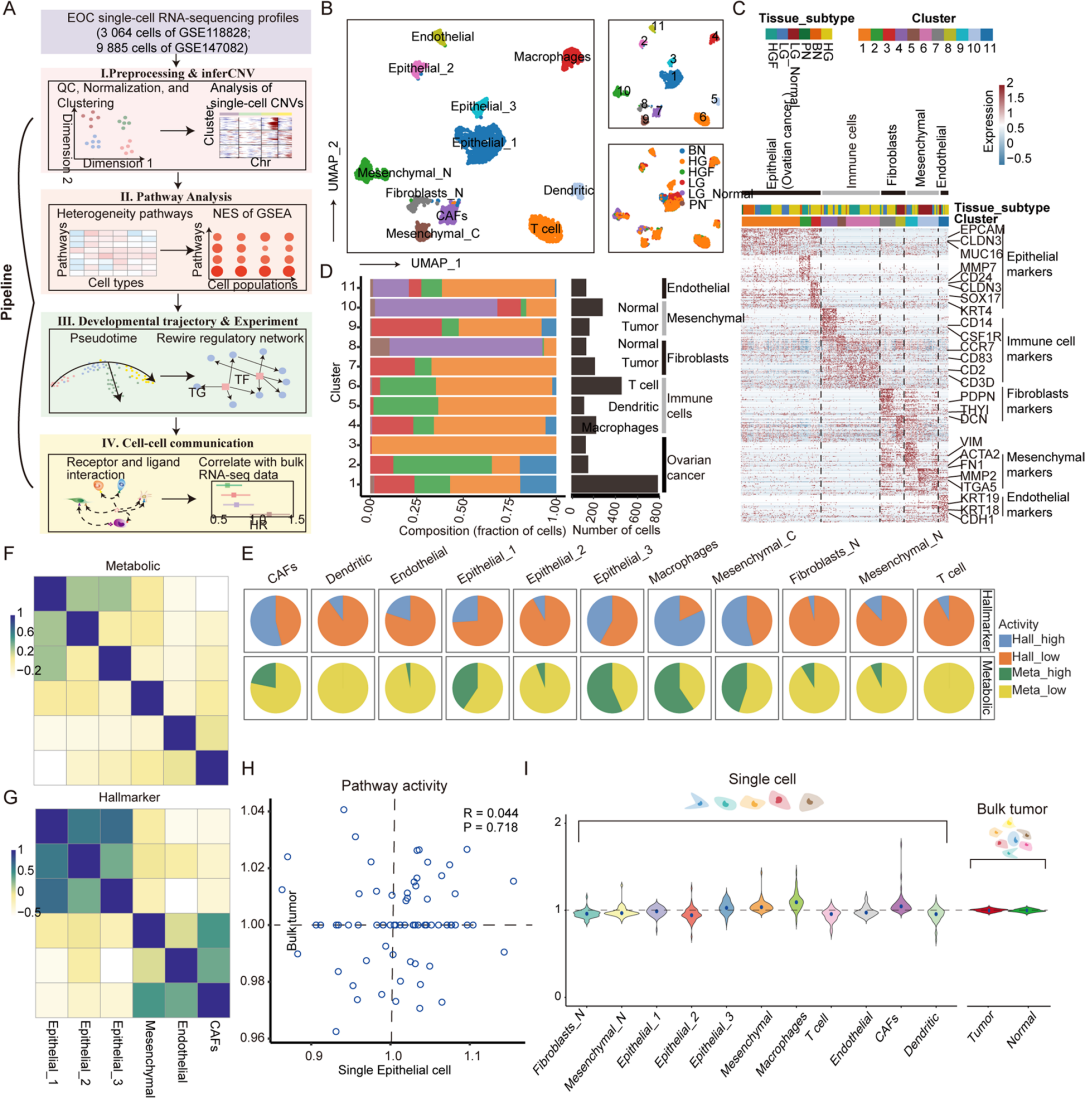
Single-cell landscape of EOC. **A** Diagram of the multiple components and workflows of the pipeline. **B** UMAP dimensionality reduction plot showing cell type annotations, clustering results including 11 clusters, and clinical typing of cells. **C** Heatmap of differentially expressed genes. Expression levels of the top 50 genes (rows) differentially expressed in each cluster (column). **D** The number of cells per cluster and total number of cells in each clinical subtype, which are distributed among the identified cell types. **E** Proportion of up- and downregulated pathways in metabolic pathways and hallmarks among cell types. **F** Correlation of cell types other than immune cells in metabolic pathway activity. **G** Correlation of cell types other than immune cells in hallmark gene sets. **H** Scatter plot comparing pathway activity between OV bulk tumours in GEO and individual malignant cells in the scRNA-seq dataset. **I** The left panel shows the distribution of pathway activity in different cell types in single-cell RNA-seq, and the right panel shows the pathway activity in bulk tumour and normal samples from GEO

We performed a systemic pipeline to analyse the single-cell RNA-seq profiles of EOC (Fig. 1A). First, the single cells after quality control of the GSE118828 cohort (Supplementary Figure S1) were classified into 11 clusters using UMAP [15] (Fig. 1B) and were annotated as seven cell types based on cell markers [11] (Fig. 1C; see Methods). Upon contacting the patient for clinical information, we found that benign, low- and high-grade serous EOC cells in Cluster 1 were clearly distributed in three regions. Moreover, the clusters of malignant epithelial cells reflected different patient sources (Supplementary Figure S2A and B). These findings suggested individual differences in tumours, which were probably related to copy number variations (CNVs) [16]; this hypothesis was confirmed by InferCNV. Clusters 1, 2 and 3, defined as epithelial cells, had a global accumulation of CNVs (Supplementary Figure S2C), suggesting the malignancy of these subsets. Furthermore, immune cells were the most abundant component in addition to malignant epithelial cells (Fig. 1D), where the immune cell content of benign, LGSOC, and HGSOC increased sequentially. Taken together, these results characterized the single-cell landscape of EOCs.

### Cell type-specific reprogramming of cancer hallmarks

Tumour development is accompanied by malignant transformation of hallmark characteristics of cancers, such as cell metabolism, signal transduction, cell proliferation and apoptosis [17]. Here, two important cancer hallmarks, metabolic reprogramming and tissue metastasis, were highlighted. The pathway activity evaluation algorithm (see Methods) was used to calculate the pathway score of each cell type. There were 69 metabolic pathways that were found to be significantly associated with different EOC cell subtypes. More than 60 pathways were highly activated (activity score > 1 and *p* value < 0.01) in at least one cell type (Supplementary Figure S3A). Similarly, there were 50 cancer hallmark gene sets that were highly activated in at least one cell type (Supplementary Figure S3B). As an example, the different activity levels of oxidative phosphorylation (OXPHOS), glycolysis, the TCA cycle, and hypoxia in the cell types were supported by direct comparison of the average expression of these pathways in each cell type (one-way ANOVA *p* < 0.01, Supplementary Figure S4), which indicated the reliability of the pathway activity evaluation algorithm. Compared with other cells, macrophages were significantly associated with more positive activation of metabolic pathways and hallmark gene sets (Fig. 1E). These upregulated gene sets were included in several parts of physiological functions, such as OXPHOS, glycolysis and EMT. Moreover, the differences in malignant epithelial cell subpopulations were reflected in metabolic activity and tumour invasion mechanisms, where type-1 epithelial cells were hypermetabolically active and had higher EMT activity, type-2 cells were moderately metabolically active, and type-3 cells were hypometabolically active (Supplementary Figure S3 and S5). Although metabolic pathway activity was strongly influenced by cell subsets (Fig. 1F), there was a high correlation in oncogenic function between the three malignant epithelial cells (Fig. 1G). This pattern was also found between CAFs and endothelial cells.

Contrasting physiological features of EOC observed at single-cell and bulk resolution contribute to the improvement of our previous understanding EOC pathogenesis. The pathway activity scores of microarray data for bulk tumour samples of EOC obtained from GEO (GSE26712) were calculated and compared with the results detected by scRNA-seq profiles. We found that only 26 metabolic pathways and 14 hallmark gene sets were upregulated in tumour samples compared to normal samples (*p* value < 0.01, Supplementary Figure S6A and B). The correlation of physiological pathway activity between bulk tumour samples and malignant epithelial cells was not significant (Pearson’s *R* = 0.044 and *p* value = 0.718 for metabolic pathways; Pearson’s *R* = 0.235 and *p* value = 0.1 for hallmark gene sets; Fig. 1H, Supplementary Figure S6 C and D), suggesting that expression specificity between cell types was masked by the fact that bulk data measure the average expression levels over a mixture of multiple cell types. Moreover, the variation in the distribution of metabolic pathway activity for single-cell data was greater than bulk data (average standard deviation of pathway activity = 0.075 for single cells compared to 0.015 for bulk tumour, Fig. 1I). Taken together, the programming of physiological function could be better reflected at single-cell resolution.

### Intratumor heterogeneity associated with TME reprogramming

**Fig. 2:**
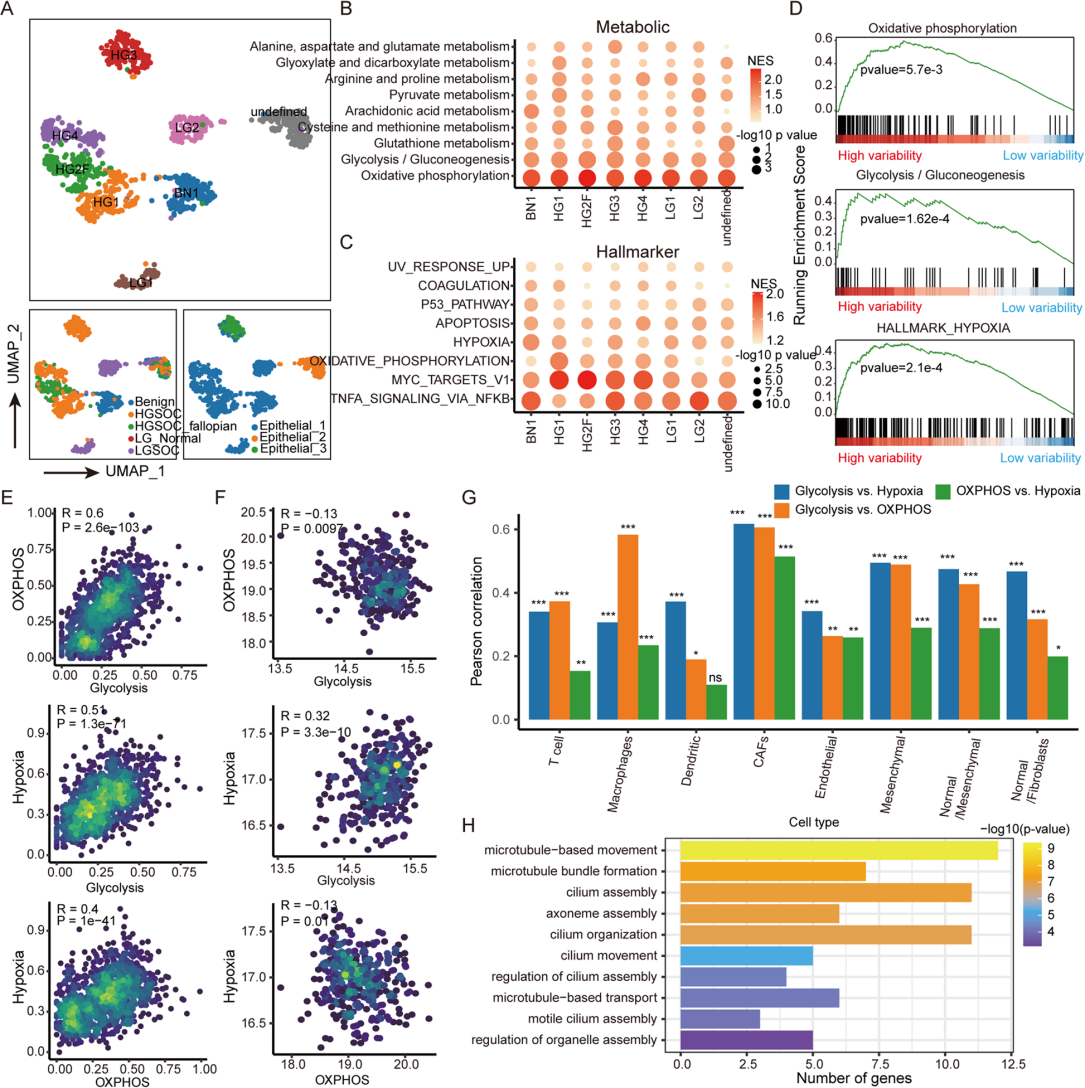
Major contributors to intratumor heterogeneity. **A** UMAP plots of malignant epithelial cell reclustering. Each cell is labelled with tissue origin, clinical typing and first clustering result. **B** Scatter plot demonstrating GSEA results for metabolic pathways weighted by principal component analysis. **C** Scatter plot demonstrating GSEA enrichment results for hallmark gene sets. **D** Ranking genes of integrated malignant tumour cells according to principal components. The plot shows GSEA enrichment results for OXPHOS, glycolytic and hypoxic gene sets. **E**, **F** Density scatter plot demonstrating glycolysis, OXPHOS activity and response to hypoxia in malignant epithelial cells based on single-cell and bulk data. The colour of the dots indicates the local density. **G** Pearson correlation between glycolysis, OXPHOS activity and response to hypoxia in other cell types of TME. The height of the bar reflects the correlation coefficient, and the number of * reflects *P*-value. The * represents *P* ≤ 0.05, ** represents *P* ≤ 0.01, *** represents *P* ≤ 0.001, and ns represents *P* > 0.05. **H** Enrichment of upregulated genes in GO from low activity cells in the OXPHOS, glycolytic and hypoxic pathways

For malignant epithelial cells, we performed reclustering analysis and identified eight clusters predominantly from seven patients (Fig. 2A), indicating individual differences in tumour cells. There was also a mixed cluster of cells overlapping with type 2 epithelial cells, suggesting that hypometabolically active malignant cells are widely distributed in EOC patients.

Reprogramming of physiological mechanisms is largely determined by variation and the environment. To explore what aspects of cellular physiological mechanisms are affected by environmental factors, GSEA [18] was used to identify metabolic and cancer hallmark gene sets enriched in genes that represent expression variation among cells (Supplementary Figure S7). We found that the OXPHOS and NFKB signalling pathways had the highest enrichment scores in most tumour clusters (Fig. 2B and C). Similarly, glycolysis and hypoxic signalling pathways also provided potential contributions to intratumor heterogeneity across these tumour clusters. The coefficient of variation (CV), standard deviation (SD), and information entropy were then used as weights for each gene accounting for variation between malignant cells to exclude potential prejudice for formula selection. Consistent with the results based on PCA (Supplementary Figure S8), these results suggest that energy metabolism and cellular resistance to apoptosis were major contributors to intratumor heterogeneity.

Furthermore, the association between energy metabolism and environmental factors such as oxygen supply was determined. Inputting the integrated gene expression matrix of malignant tumour cells, the GSEA results showed that OXPHOS, glycolysis and hypoxia were significantly enriched in ranked genes (Fig. 2D). The hypoxic signals (hypoxia inducible factor, HIF) that indirectly reflect the oxygen content of cells were evaluated. We found a high correlation between glycolysis and hypoxia (Pearson’s *R* = 0.51 for epithelial cells, Fig. 2E). Moreover, the activity of OXPHOS was significantly correlated with glycolysis (Pearson’s *R* = 0.62) and the response to hypoxia (Pearson’s *R* = 0.43). Notably, we did not detect a strong correlation between OXPHOS and glycolysis (Pearson’s *R* = -0.13) or between OXPHOS and hypoxia (Pearson’s *R* = -0.13) using bulk RNA-seq data from the TCGA (Fig. 2F). In addition to malignant epithelial cells, we also evaluated the relationship among OXPHOS, glycolysis and hypoxia in other cell types of TME (Fig. 2G), indicating the specific relationship between energy metabolism and hypoxia was evident in CAFs at single-cell resolution. For cells with low activity in energy metabolism, the upregulated genes of these cells were significantly enriched in microtubule-based movement (Fig. 2H), suggesting that the subpopulation of malignant epithelial cells is related to tissue invasion. Taken together, energy metabolism was the major contributor to tumour heterogeneity.

### Reprogramming of energy metabolism during EMT

**Fig. 3:**
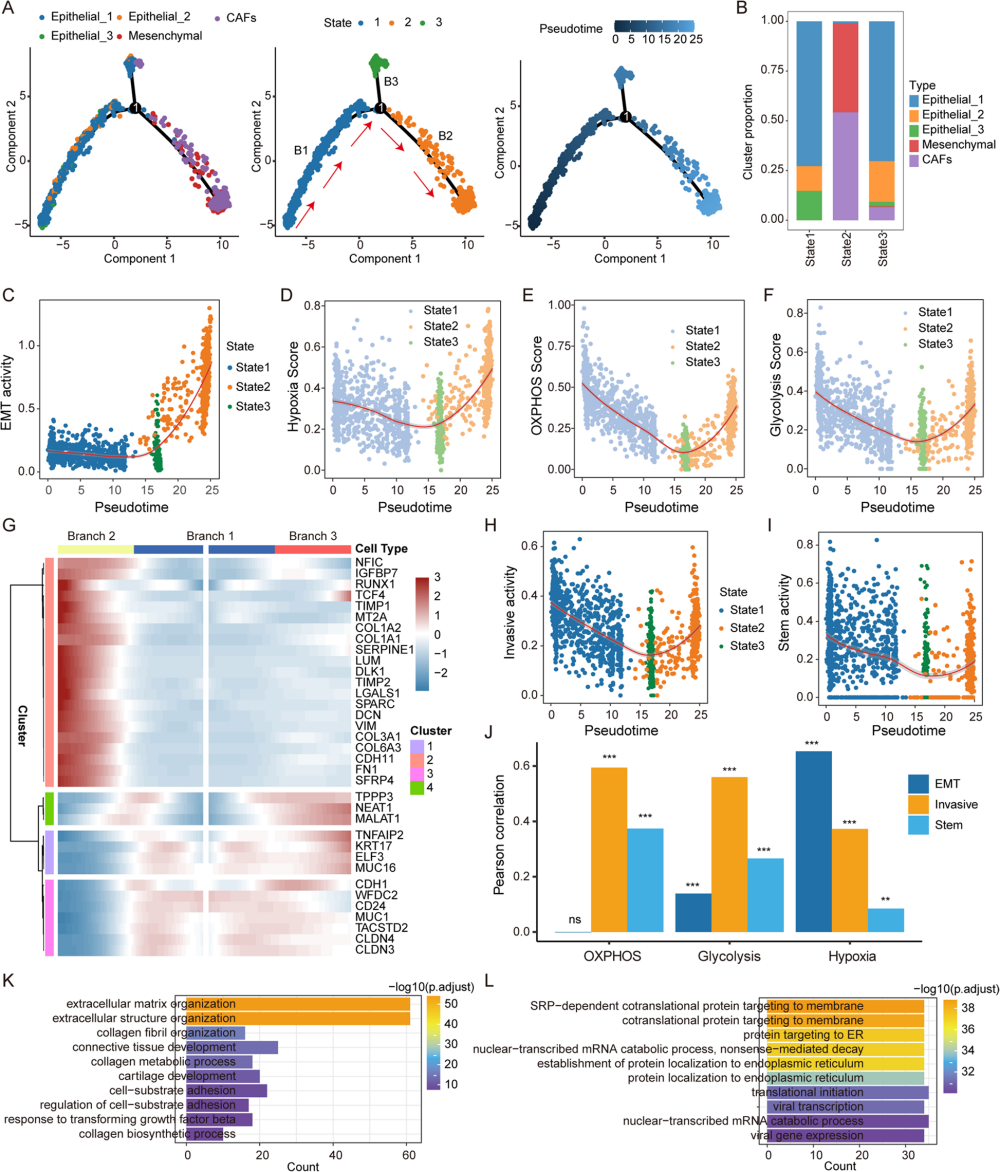
Specific energy supply during EMT. **A** Developmental trajectory of cells in two dimensions. Cells are coloured according to cell type (left), state (middle), and pseudotime (right). The red arrows indicate the defined "epithelial-mesenchymal path". **B** The number of cells per cell type in each developmental state. **C** Variations in EMT pathway activity with pseudotime. **D**-**F** The pathway activity of OXPHOS, glycolytic and hypoxic distribution with pseudotime. **G** The heatmap shows the branch-dependent genes at branch point 1. The centre of the heatmap indicates branch B1, the left indicates branch B2, and the right indicates branch B3. **H** The invasive activity distribution with pseudotime. **I** The stem activity distribution with pseudotime. **J** Bar graphs reflect the Pearson correlation between OXPHOS, glycolysis and hypoxia pathway activity and EMT, invasion and stemness pathway activity. The height of the bar reflects the correlation coefficient, and the number of * reflects *P*-value. **K**-**L** Enrichment of up- and downregulated genes associated with pseudotime in the GO term

There was a certain relationship between the energy metabolism of tumours and cell invasion. However, a temporal map of energy metabolism during cell invasion is lacking. Furthermore, the pseudo-developmental trajectory of epithelial cells to mesenchymal cells was simulated using Monocle2 (Fig. 3A). The cells were grouped into three branches (defined as “B1”, “B2” and “B3”) with different states. We found that the three epithelial cell subtypes were mainly concentrated in state 1 and state 3, while mesenchymal cells and CAFs were concentrated in state 2 (Fig. 3B). The EMT pathway activity gradually increased with pseudotime development and reached its highest level at state 2 (Fig. 3C). Moreover, we found that the scores of OXPHOS, glycolysis and hypoxia increased from branch 1 to branch 2 during the EMT process (Fig. 3D-F), which was consistent with the above studies suggesting that cells with lower energy metabolism had potential for invasion. Several branching-dependent genes were identified from branch 1 to branch 2, and most of them were related to the tissue invasion and energy metabolism (Fig. 3G). Moreover, cell invasiveness and stemness followed similar trends to energy metabolism during the EMT process (Fig. 3H and I). We next assessed the correlation of hypoxia-associated energy metabolism (OXPHOS, glycolysis, and hypoxia) with the physiological function (EMT, invasion, and stemness) of malignant cells. We found that OXPHOS and glycolysis were more highly correlated with cell invasiveness (Pearson’s = 0.59 for OXPHOS, Pearson’s = 0.56 for glycolysis; Fig. 3J). In addition, 810 EMT-related genes (586 positively and 224 negatively; |R|>0.2) were identified based on pseudotime analysis. GO functional enrichment results showed that positive EMT-related genes were mainly enriched in the extracellular matrix and cell adhesion, which may provide the requirements for cell invasion (Fig. 3K), and negative EMT-related genes were associated with the encoding of membrane proteins (Fig. 3L). These results indicate that energy metabolism associated with hypoxia plays an important role in tumour tissue invasion.

### Prognostic markers of cell invasion in EOC

**Fig. 4:**
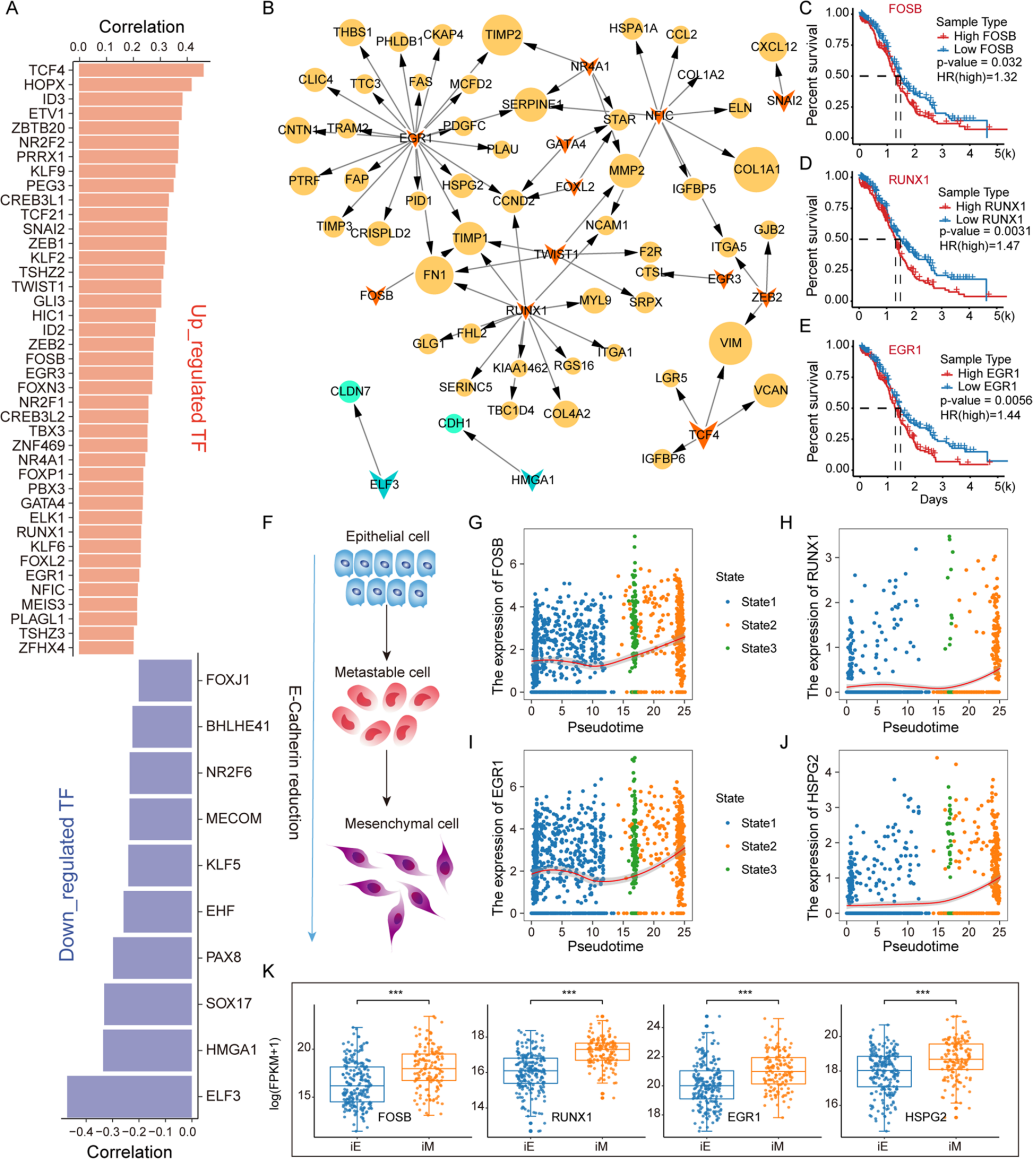
Critical factors boost EMT in EOC. **A** Up- and downregulated TFs and their Pearson correlation coefficients with pseudotime. **B** Transcriptional regulatory network constructed using temporal-associated genes. The diamond represents the TF, and the circle represents the target gene. The size of the graph is determined by the coefficients of positively (yellow) and negatively (green) genes. **C**-**E** Survival curves of FOSB, RUNX1 and EGR1 gene expression in the TCGA-OV dataset. **F** Schematic diagram of the reversal of epithelial cells with reduced E-cadherin into mesenchymal cells. **G**-**J** Variation in the expression levels of FOSB, RUNX1, EGR1, and HSPG2, which are transcription factors and target genes, with pseudotime. **K** Box plot of gene expression variations, including FOSB, RUNX1, EGR1, and HSPG2, between iE and iM samples of bulk RNA-seq from TCGA

The driver genes related to EMT pseudotime were identified based on the molecular regulatory network. In the transcriptional regulatory network, we identified 41 positive-TFs and 10 negative-TFs from EMT-related genes (Fig. 4A), which targeted 50 positive-target genes and two negative-target genes (Fig. 4B). We found that FOSB and RUNX1, as important drivers of tumour proliferation and invasion [19, 20], were significantly associated with the OS of EOC patients (Fig. 4C and D) and upregulated during EMT (Fig. 4F-H). It has been demonstrated that EGR1 can regulate angiogenesis to promote tumour cell invasion [21], which is upregulated in the EMT process and regulates the target gene HSPG2 (Fig. 4F-J) to promote EMT. Notably, high expression of both EGR1 and HSPG2 was associated with poorer OS in EOC patients (Fig. 4E, Supplementary Figure S9A). In addition, HMGA1 downregulated the expression of CDH1, encoding E-cadherin (Supplementary Figure S9B and C). Furthermore, FOSB, RUNX1, HSPG2, EGR1, HMGA1 and CDH1 were significantly differentially expressed between invasive epithelial (iE) and invasive mesenchymal (iM) samples defined from TCGA-OV data [22, 23] (Fig. 4K and Supplementary Figure S10), suggesting that these factors driving the EMT process identified at single-cell resolution are reliable.

Furthermore, independent single-cell sequencing data of EOC were used to validate the above results. We performed cell clustering, cell annotation and trajectory analysis of the EOC cells in independent dataset (GSE147082) (Supplementary Figure S11A). We found that different groups of epithelial cells and mesenchymal cells were mainly concentrated in separate branches and cellular states with continuously increased pseudotime (Supplementary Figure S11B-D). The EMT pathway activity was found to be increased from state 2 to states 1 and 3 (Supplementary Figure S11E). Furthermore, the expression of TFs and their targets, which were identified in the above analysis (such as EGR1, FAS and HSPG2), was also found to be increased from state 2 to states 1 and 3 (Supplementary Figure S11F-I). We identified the presence of multiple transcriptional regulatory pairs and highlighted the positive correlation between EGR1 and its target genes HSPG2 and FAS and EMT activity (Supplementary Figure S11J). These results suggested that the similar EMT development trajectory of EOC and driver genes can be recaptured based on an independent dataset.

### Knockdown of EGR1 inhibits ovarian cancer cell migration and invasion

**Fig. 5:**
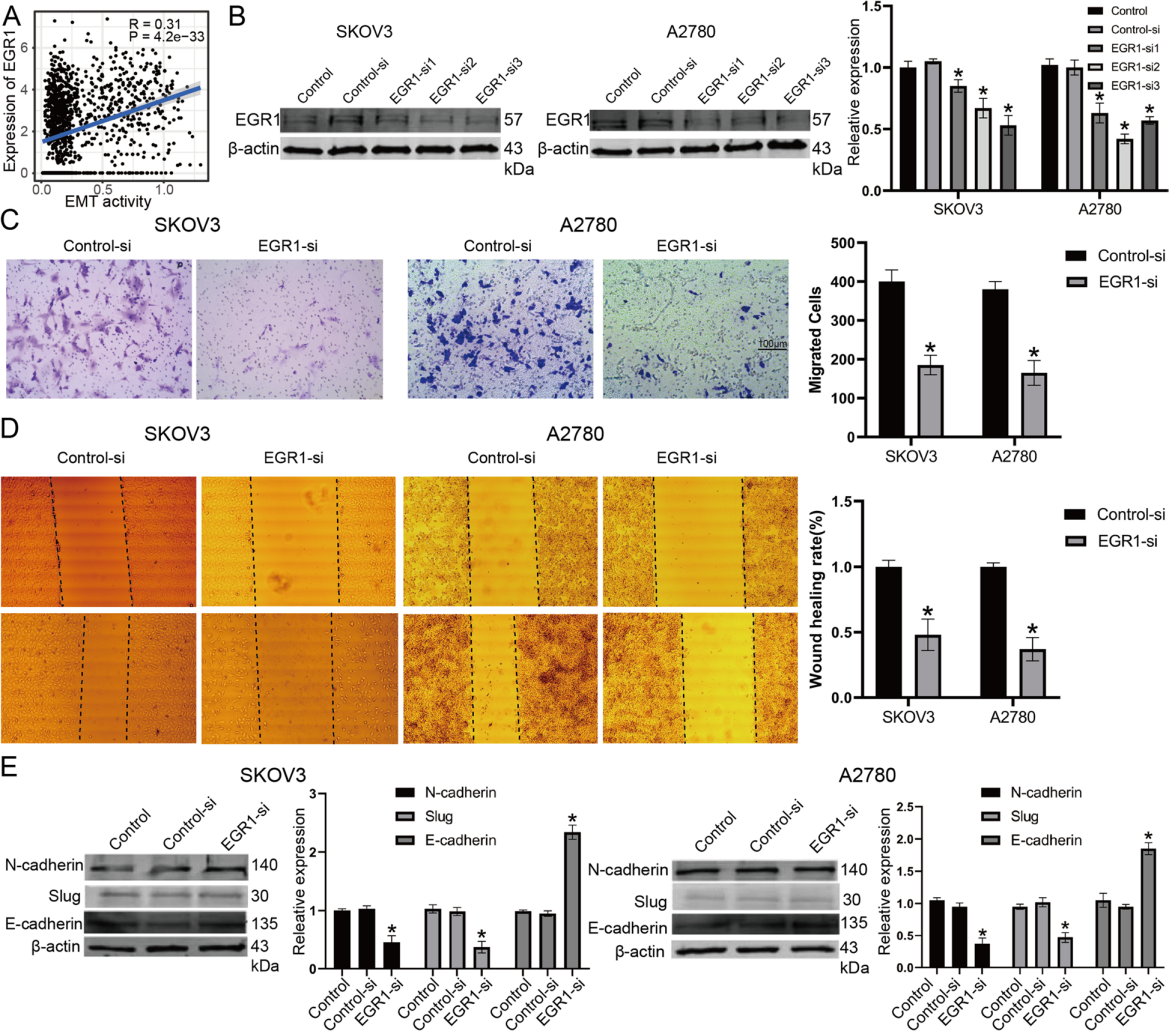
Knockdown of EGR1 inhibits ovarian cancer cell migration and invasion. **A** A positive correlation was also observed between EMT activity and EGR1 expression in the GSE118828 dataset. **B** Knockout efficiency of EGR1 in SKOV3 and A2780 cell lines. **C** Evaluation of the effect of EGR1 on the migration and invasion of ovarian cancer cells by Transwell analysis. **D** Evaluation of the effect of EGR1 on the migration and invasion of ovarian cancer cells by wound healing analysis. **E** Significantly increased expression of E-cadherin and decreased expression of N-cadherin and Slug were observed after EGR1 silencing

The TF EGR1 regulated most EMT-related genes (Fig. 4B) and exhibited significant prognostic efficiency for patient survival in EOC datasets (Fig. 4E and Supplementary Table S1). A positive correlation was also observed between EMT activity and EGR1 expression (Fig. 5A). Furthermore, experimental analysis was performed by employing two ovarian cancer cell lines, SKOV3 and A2780. Western blotting was performed to show the knockout efficiency of EGR1 in SKOV3 and A2780 cells (Fig. 5B). We evaluated the effect of EGR1 on the migration and invasion of EOC cells by Transwell and wound healing assays (Fig. 5C-D). The migration and invasion capacities of SKOV3 and A2780 cells were significantly decreased compared to those of the control group. On the other hand, we tested EMT-related proteins in SKOV3 and A2780 cells after treatment with EGR1 inhibition (Fig. 5E). Our data showed that silencing EGR1 significantly increased the expression of E-cadherin. The N-cadherin and Slug proteins were downregulated in the EGR1-si group. These results indicate that silencing EGR1 suppressed invasion in ovarian cancer cells.

### Inhibition of EGR1 expression decreases the expression of FAS and HSPG2

**Fig. 6:**
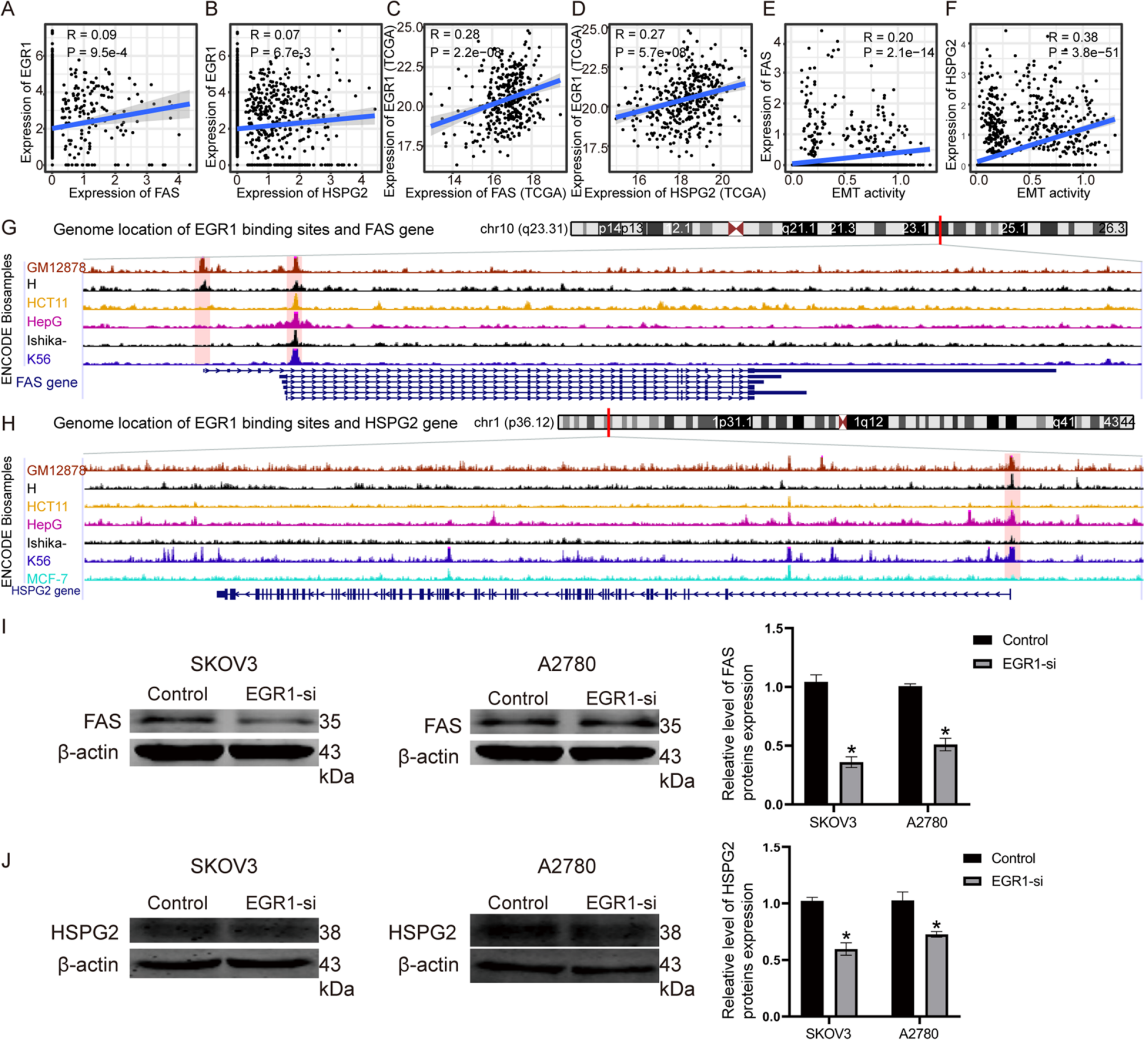
Suppressed EGR1 Decreases Expression of FAS and HSPG2. **A**-**D** A positive correlation was observed between EGR1 and its targets FAS and HSPG2 in the GSE118828 and TCGA datasets. **E**-**F** The FAS and HSPG2 expression levels were positively correlated with EMT activity. **G**-**H** Enriched EGR1 sequencing read peaks were found in the FAS and HSPG2 transcription factor binding regions. **I**-**J** The protein expression levels of FAS and HSPG2 were markedly downregulated in the EGR1-si group in SKOV3 and A2780 cells

Next, the regulatory mechanism of EGR1 was investigated. The expression of two EGR1 target genes, FAS and HSPG2, was positively correlated with EGR1 based on the single-cell expression profile and TCGA bulk dataset (Fig. 6A-D). Within the TME of EOC, cells with high FAS and HSPG2 expression levels exhibited increased EMT activity (Fig. 6E-F). To further confirm the transcriptional relationship between EGR1 and downstream target genes, we explored the chromatin immunoprecipitation sequencing (ChIP-seq) experiment dataset from ENCODE. Enriched EGR1 sequencing read peaks were found in the FAS and HSPG2 transcription factor binding region across different biosamples, indicating a direct transcriptional relationship between EGR1 and its targets (Fig. 6G-H). The experiment demonstrated that FAS and HSPG2 expression at the protein level was markedly downregulated in the EGR1-si group in SKOV3 and A2780 cells compared with the NC-si group (Fig. 6I-J). These results indicated that EGR1 decreases the expression of FAS and HSPG2 and is associated with EMT in EOC.

### Cell‒cell communication reveals the pro-invasive effect of CAFs

**Fig. 7:**
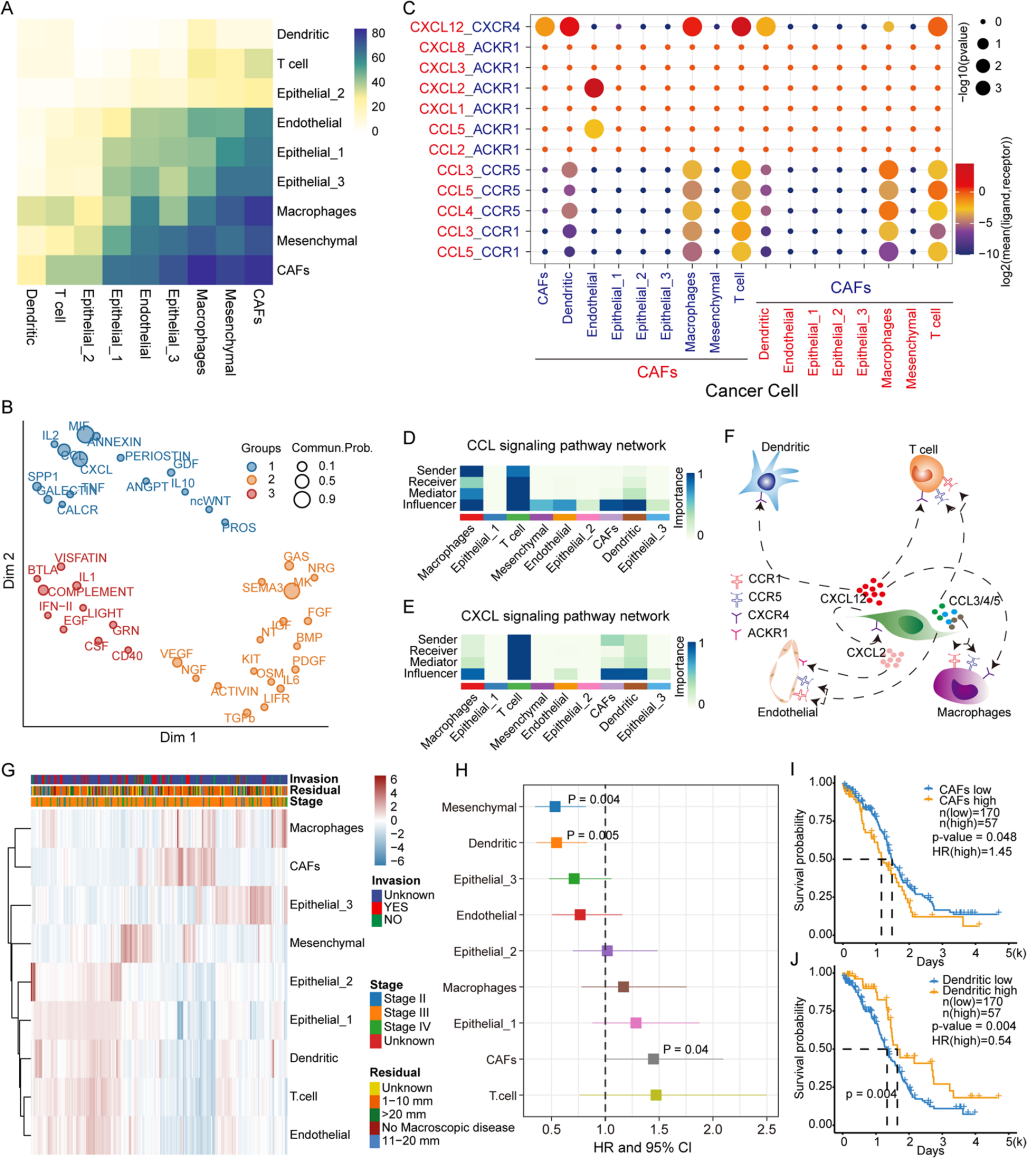
Cell‒cell communication reveals the pro-metastatic effect of CAFs. **A** The heatmap shows the number of potential ligand‒receptor pairs between cell populations predicted by CellChat. **B** Signalling pathways are clustered into 3 categories based on their function. **C** The bubble plot shows the ligand‒receptor pairs of CXCL and CCL. **D** The amount of communication, including sender, receiver, mediator and influencer, between each cell type in the CXCL signalling pathway. **E** Same as in D but for the CCL signalling pathway. **F** Predictive signalling factor regulatory network centred on CAFs. **G** Heatmap of cell abundance for each sample predicted based on bulk RNA-seq data by CIBERSORTx. Shown are Row z scores. **H** COX risk regression to assess the association between relative cell abundance and patient survival from bulk RNA-seq. **I**-**J** Kaplan‒Meier curves of patient survival in relation to CAFs and dendritic cells

The 42 signalling pathways that enable cell‒cell communication between nine cell subsets in tumour tissue were identified by CellChat [24], and the amount of signal emission in the three epithelial cell clusters correlated with metabolic pathway activity (Supplementary Figure S12). Notably, CAFs exhibited the most communication with other cell subsets and had the strongest interactions with mesenchymal and macrophage cells (Fig. 7A). To explore the functions of these signalling pathways, we clustered the above 42 signalling pathways into three categories related to growth factors and invasion functions (Fig. 7B). Furthermore, we identified CCL and CXCL family ligand‒receptor pairs associated with tumour invasion (Fig. 7C-E). The CAFs highly expressed the ligand CXCL12 (Fig. 7C). We found that the receptor CXCR4 was highly expressed in immune cells such as dendritic cells, T cells and macrophages. CCL5 and CXCL2, secreted by CAFs, could interact with ACKR1, which is highly expressed on the surface of endothelial cells (Fig. 7C and F). The role of CAFs in tumour proliferation and invasion revealed that EOC patients have individual clinical phenotypes that may be related to the cellular composition of the TME. The fraction of the nine cell subsets in the OV samples from the TCGA was calculated using cibersortX (Fig. 7G). Correlating the cell fraction data with clinical information, we noted that some stage III patients had a higher fraction of CAFs but a lower fraction of lymphocytes. The accumulation of CAFs was also associated with poorer OS, suggesting that CAFs play an important role in tumour progression (Fig. 7H and I). However, the accumulation of dendritic cells was associated with better OS, suggesting that it may be a protective factor for EOC (Fig. 7J). Taken together, these results highlight that CAFs promote EOC progression and are related to patient prognosis.

## Discussion

In this study, we re-analysed two cohorts of scRNA-seq datasets of EOC and characterized the relationship between TME reprogramming and tissue invasion. In the TME, macrophages and type 1 malignant epithelial cells had global metabolic pathway activation. The activity of energy metabolism contributed to the intratumor heterogeneity of malignant epithelial cells. Furthermore, we simulated EMT trajectories using pseudotime analysis, revealing that hypoxia, OXPHOS, and glycolysis were closely related to the EMT process, which is also induced by key factors identified by transcriptional regulation. The key signals of malignant epithelial cell invasion based on cellular communication were identified, and the components of CAFs in TME were highlighted.

Malignant epithelial cells were defined by cell markers and CNVs, and individual differences were also observed (Fig. 1B, C; Supplementary Figure S2A-C), which may be caused by the patient’s unique physiological environment, including location-specific characteristics and nutrient supply [25]. The overall proportion of immune cells in each clinical subtype of tumour may reflect the different efficiencies of immune depletion [11]. We found that HGSOC had the highest T cell infiltration compared to LGSOC and benign (Fig. 1D), which may be a plausible explanation for why high-grade serous ovarian cancers are more suitable for immune-targeted therapy [26].

The relationship between energy metabolism and oxygen supply is intriguing. The TME of ovarian cancer underwent a reprogramming of metabolic and physiological functions at single-cell resolution and differed from that in bulk tumour studies [27] (Fig. 1E-I), and energy metabolism plays a key role in the intra-tumour heterogeneity (Fig. 2A-D). The positive correlation between hypoxia and glycolysis is consistent with previous studies demonstrating that hypoxia activates glycolysis [28, 29]. Hypoxia positively correlates with OXPHOS (Fig. 2E-G), contradicting the phenomenon that OXPHOS is suppressed in tumours by comparing the expression of metabolic genes between bulk tumours and normal tissues [30, 31]. This may be due to the greater competition for oxygen by tumour cells. We found that a subpopulation of cells with lower OXPHOS and glycolytic activity significantly activated cell invasion-related pathways (Fig. 2H), which suggested energetic metabolic reprogramming of tumour cell invasion.

We used a reverse graph embedding method to simulate the EMT process and found the activity trajectory of energy metabolism and stemness (Fig. 3A-I). Hypoxia-mediated energy metabolism significantly correlated with cell invasiveness (Fig. 3J-L). Several cell line experiments have demonstrated that hypoxia induces EMT in tumour cells [32, 33], which is consistent with our findings. Through public data and experimental verification, we found that inhibiting the expression of EGR1 can regulate the invasion of tumour cells (Fig. 4), and the decreased expression of EGR1 significantly upregulated the expression of E-cadherin (Fig. 5) and downregulated the targeted genes FAS and HSPG2 (Fig. 6). Moreover, cytokines play an important role in reregulating tumour cell invasion and immune microenvironment. Previous studies suggested that CXCL12 from CAFs could combine with the receptor CXCR4 in epithelial cells to induce tissue invasion [14] (Fig. 7A-F). The CCL5/CXCL2-ACKR1 pairs could stimulate vascular endothelial cells to promote leukocyte infiltration [34, 35]. Importantly, CAFs acted as important signal transmitters in the TME regulating tumour progression and patient prognosis (Fig. 7G-J).

To further evaluate the regulatory roles of driver genes related to the EMT process, we considered the ceRNA competition mechanism and constructed a ceRNA regulatory network involving four lncRNAs (two positively correlated and two negatively correlated for EMT process) and nine corresponding mRNAs (seven positively correlated and two negatively correlated for EMT process, Supplementary Figure S13A). We found that GALNT1 expression is upregulated during EMT (Supplementary Figure S13B) and has been confirmed to promote tumour proliferation and invasion in colorectal cancer [36]. With the gradual decrease in the expression of E-cadherin, USP53, which was upregulated in tumour cell (Supplementary Figure S13C), could promote apoptosis through FKBP51-AKT1 signalling [37]. This finding is consistent with previous studies showing that cancer cells lacking E-calcineurin are more likely to undergo “apoptosis”, although they are more easily subjected to aggressive growth [38]. We found that the gene RHOBTB3, which regulates the expression of HIFα [39], was upregulated during the EMT process (Supplementary Figure S13D).

## Conclusions

In summary, this study provides a global landscape of TME reprogramming and identifies several genes related to EOC tissue invasion at single-cell resolution. Although we analysed the physiological mechanisms of EOC only in terms of metabolism and cancer hallmark gene sets, this is also sufficient to explain tumour proliferation and invasion. The results of this study may provide a more precise theoretical basis for the treatment strategy of EOC.

## Methods

### Data collection and pre-processing

The single-cell RNA-seq profiles for EOC were collected from the Gene Expression Omnibus (GEO; https://www.ncbi.nlm.nih.gov/gds) under accession number GSE118828. The profiles were taken from different tissues of nine patients, andeighteen sequencing files were obtained, containing 3 064 single cells. The samples were derived from different types of ovarian tissue: one from peritoneal, one from benign, one from normal ovarian tissue, three from low-grade serous ovarian cancer (LGSOC), twelve from high-grade serous ovarian cancer (HGSOC) (Supplementary Table S2). After quality control (Supplementary Figure S1), the expression matrix of 2 794 cells was normalized using Scran [40] due to the low sequencing depth. The normalized matrix was used in principal component analysis (PCA) and clustering analysis using UMAP implemented in Seurat [41]. Marker genes for specific cell types collected from CellMarker [42] (http://biocc.hrbmu.edu.cn/CellMarker/) and published literature [11] were used for cell annotation. Independent single-cell RNA-seq profiles (GSE147082) including 9 885 cells of six samples from six EOC patients were collected. The five of six samples were defined as serous ovarian cancer (Supplementary Table S3) and used as the validation dataset. The microarray data (GSE26712) and bulk RNA-seq data (TCGA-OV) of serous ovarian cancer were collected for comparative analysis. The clinical information of these samples was also downloaded and the statistical results were showed in Supplementary Table S4 and S5. Epithelial serous ovarian cancer patients were mainly concentrated in stage III and the five-year survival rate was less than 20%.

### Cell annotation

Based on previous studies [11], we annotated the cell types of these 11 cell clusters as epithelial cells (Clusters 1, 2 and 3 marked by KRT4, EPCAM, MMP7, CLDN3, CLDN4, CD24), macrophages (Cluster 4 marked by CD14, AIF1, CSF1R, CD163), dendritic cells (Cluster 5 marked by CCR7, CD83), T cells (Cluster 6 marked by CD2, CD3D), fibroblasts including normal and tumour cells (Clusters 7 and 8 marked by PDPN, DCN, THY1, MMP2), mesenchymal cells including normal and tumour cells (2 Clusters 9 and 10 marked by PDPN, DCN, THY1, MMP2), and endothelial cells (Cluster 11 marked by KRT18/19, CDH1).

### Calculation of the pathway activity

The weighted relative pathway activity algorithm [25] was used to evaluate the activity of metabolic and hallmark pathways in each major cell type. First, the expression of a metabolic or hallmark gene in each cell type was defined by the mean expression level. Second, we quantified the relative expression of a metabolic or hallmark gene in a specific cell type by comparing it to the average expression level of the gene in all cell types. Finally, the activity score for the pathway in a specific cell type was then defined as the weighted average of the relative expression of all genes included in this pathway. Furthermore, the weighting factor, used to eliminate commonalities between eachmetabolic pathway, for a gene was defined as the reciprocal of the number of pathways that include this gene.

We deleted genes that had low expression levels or a high dropout rate in the pathway to avoid distortion of activity scores. To verify the statistical significance of the pathway activity score, we randomly swapped cell labels 5000 times to construct a null distribution of pathway activity scores. Furthermore, the *p* value was calculated to assess whether the activity of this pathway was significantly higher or lower than average. In addition, the gene lists of metabolic pathways and hallmarks were obtained from the molecular signatures database (MSigDB [43], http://www.broadinstitute.org/msigdb). Only pathways and hallmarks with more than 5 genes that were detected in the sequencing results were kept for further analysis.

### Analysis of intercellular heterogeneity

PCA was performed on the normalized RNA-seq profiles. For each gene in metabolic pathway and hallmark, we defined the weight of a gene as the sum of the absolute values of the loadings of this gene on the top PCs that explains at least 85% of the overall variation of the single-cell RNA-seq or bulk microarray profiles. Genes were sorted in descending order according to their weights, and gene set enrichment analysis (GSEA) was used to identify metabolic pathways and hallmark gene sets (collected from MSigDB) enriched in the ranked genes. Moreover, the coefficient of variation (CV), standard deviation (SD), and information entropy were also used to interpret intercellular heterogeneity.

### Developmental trajectory analysis

To explore the process of epithelial mesenchymal transition (EMT), epithelial cells, mesenchymal cells and CAFs were extracted for the construction of pseudotime developmental trajectories using Monocle [44] (v2.16.0). The break points and branches were marked in the developmental trajectory, where cells in the same segment of the trajectory were defined to have the same state.

### Construction of regulatory networks for EMT-related genes

Pearson correlation analysis was used to evaluate genes related to pseudotime. We defined genes with |P|>0.2 [45] as EMT-related genes. The expression variation direction of transcription factors (TFs) and corresponding target genes (TGs) included in EMT-related genes were supposed to be a regulatory pair in the pseudotime process, that is, ${P}_{tf} \text{x} {P}_{tg}>0$. Human transcription factor and transcriptional regulatory interaction data were retrieved from AnimalTFDB [46] 3.0 (http://bioinfo.life.hust.edu.cn/AnimalTFDB/) and TRRUST [47] v2 (www.grnpedia.org/trrust), respectively. Furthermore, we found that the expression level was positively correlated for each lncRNA‒mRNA pair due to the ceRNA network mechanism. The lncRNA and mRNA relationship pairs in the ceRNA network were acquired from starBase [48] available at http://starbase.sysu.edu.cn/.

### Cell‒cell communication analysis with CellChat

CellChat [24] is an R-based computational analysis tool developed by Suoqin Jin et al. that analyses intercellular communication at the molecular level. All tumour cells that were defined as nine cell subsets were used to explore the intercellular signalling interaction network using CellChat. Interaction pairs that belonged to signal transduction pathways and had *P* values < 0.05 were applied to explore the relationship between cell types in the tumour microenvironment.

### Connection to public datasets

Bulk RNA-seq and phenotype data of EOC deposited in the TCGA were downloaded from UCSC XENA (https://xenabrowser.net/datapages/). Then, the CIBERSORTx [49] algorithm, which is an analysis tool for abundance estimation of member cell types in mixed cell populations developed by Newman et al., was used to calculate the cellular fraction of each sample. Subsequently, bulk samples were categorized into high and low according to three-fourths of the fraction of each cell type. The univariate Cox regression model was used to estimate cell subsets in relation to the overall survival (OS) of EOC patients. Kaplan‒Meier analysis was performed to estimate the OS curves.

### Reagents and cell cultures

Antibodies against EGR1 and EMT-related antibody kits (E-cadherin, N‐cadherin, and slug) were purchased from Cell Signaling Technology (Danvers, MA). Two human ovarian cancer cell lines (SKOV3 and A2780) were used in our study and their tissue origin information was presented in Supplementary Table S6. The SKOV-3 and A2780 cell lines were purchased from American Type Culture Collection (ATCC, South San Francisco, CA). All experiments were performed with mycoplasma-free cells. Cells were cultured in Dulbecco’s modified Eagle’s medium (DMEM; Life Technologies, Grand Island, NY) with 10% foetal bovine serum (Life Technologies) and were maintained in a humidified 5% CO2 incubator at 37 °C. All human cell lines have been authenticated using Short Tandem Repeat (STR) or SNP profiling within the last three years.

### Wound healing assay

Cells transfected with si-NC or si‐EGR1 were seeded into six‐well culture plates with serum‐containing medium and were cultured until the cell density reached 90‐95% confluence. An artificial homogeneous wound was created by scratching the monolayer with a sterile 200 µL pipette tip. After scratching, the cells were washed with PBS and then cultured with serum‐free DMEM for 48 h. Images of cells migrating into the wound were captured at 0 and 48 h using a microscope (EVOS, USA). The assay was performed in triplicate.

### Cell migration assay

Cell migration and invasion abilities were assessed by the Transwell assay. Polycarbonate transwell filters with 8.0-µm pores were inserted over the lower chambers. A total of 5 × 104 cells suspended in 2% FBS were plated on the insert chamber supplemented with 600 µl of 10% FBS medium. After 12 h, cells were fixed and permeabilized in propyl alcohol for 15 min and then stained with 5% crystal violet stain overnight at 4 °C. The number and morphology of cells were observed under an inverted microscope.

### Western blotting

Total protein was extracted by using a RIPA kit. The protein was separated by SDS‒PAGE (12.5%), transferred to an NC membrane, and later blocked with 5% nonfat milk for two hours at room temperature (Thermo Fisher). Then, the membrane was incubated with primary antibodies against E-cadherin, slug, N-cadherin, HSPG2, FAS and EGR1 at 4 °C overnight. Next, the membrane was incubated with a secondary antibody to HRP-labelled Beyotime, Beijing. Blots were viewed with electrochemiluminescence (ECL) chromogenic substrate.

### Statistical analysis

All statistical analyses and graph generation were performed in R (version 4.0.2).

### Supplementary Information

**Additional file 1: Figure S1.** (A) The histogram displays the distribution of counts detected per cell. The subplot in the upper right corner shows the distribution of the fraction of counts less than 3,000. Since here is a peak at count 700 and there are very few cells with counts greater than 20,000, the threshold is set to 500 and 15000. (B) The histogram displays the distribution of the number of genes detected per cell. There is a small peak at 300 genes (noise peak) and the threshold is set to 200. (C) The counts detected for each cell are plotted from high to low on the rank plot. 500 is the screening threshold since there is a rapidly decreasing inflection point at a counts count of 500. **Figure S2.** EOC cell distribution based on UMAP cluster plot. (A) The distribution of tumor and normal tissue cells. Each cell is labeled according to its origin. (B) The distribution of tumor cell states. (C) Copy number variations (CNVs) for per cell evaluated by InferCNV. Two normal-sample clusters and four stromal cell clusters were used as control group. **Figure S3.** Metabolic pathways and hallmark activity scores for cell subsets. (A) Metabolic pathway activity in each cell types. Values with low to high and statistically insignificant pathway activity (random permutation test *p* < 0.01) are shown as blank. (B) Hallmark activity in each cell types. **Figure S4.** The pathway and cancer hallmark activity characterization of different cell clusters. The P-values were calculated using one-way ANOVA. (A) The Glycolysis/Gluconeogenesis pathway activity status in different cell types. (B) The TCA cycle pathway activity status in different cell types. (C) The Oxidative phosphorylation pathway activity status in different cell types. (D) The HALLMARK_HYPOXIA pathway activity status in different cell types. **Figure S5.** Distribution of metabolic pathway activity scores in three clusters of epithelial cells. The difference in metabolic activity between the two is evaluated by Student's t test. **Figure S6.** (A) Metabolic pathway activity in tumor and normal samples of bulk RNA-seq. White represents insignificant pathway activity scores (random permutation test *p* > 0.01). (B) The same as in A but for hallmark gene sets. (C) Scatter plot comparing hallmark gene sets activity between OV bulk tumors in TCGA and individual malignant cells in the scRNA-seq dataset. (D) The left panel shows the distribution of hallmark gene sets activity in different cell types in single-cell RNA-seq, and the right panel shows the hallmark gene sets activity in bulk tumor and normal samples from TCGA. **Figure S7.** (A) Explanation of variance of principal components (PC) from principal component analysis (PCA) of metabolic gene expression levels in eight malignant epithelial cell clusters. Top PCs accounting for 80% of the variance are highlighted in purple. (B) The same as in A but for hallmark gene expression levels.**Figure S8.** The scatter plot showing GSEA enrichment results for metabolic pathways and hallmarks. (A-C) The metabolic pathways were weighted by coefficient of variation, standard deviation and entropy, respectively. (D-E) The hallmarks were weighted by coefficient of variation, standard deviation and entropy, respectively. **Figure S9.** (A) Survival curves comparing high and low HSPG2 gene expression with patient overall survival (OS). (B-C) Variation of HMGA1 and CDH1, which are transcriptional regulatory pairs, expression levels with pseudo-time. **Figure S10.** Box plot of genes including GALNT1, HMGA1, CDH1, USP53, and RHOBTB3 expression variations between invasive epithelial (iE) and invasive mesenchymal (iM) samples of bulk RNA-seq from TCGA. **Figure S11.** Validation of EMT process using an independent single-cell data (GSE147082). (A) The EOC cells were grouped into 13 clusters contain different cell types. (B-D) The epithelial cells and mesenchymal cells were mainly concentrated in separate branches and cellular states with continuously increased pseudo-time. (E-H) Increased EMT pathway activity, ERG1, FAS, and HSPG2 was found from state 2 to state 1 and 3. (F) Heatmap shows the branch-dependent genes at branch point 1. (G) Correlation heatmap between TF-target relationships and EMT activity. **Figure S12.** Contribution of nine cell types in ovarian cancer tissue communication. Horizontal coordinates are cell types and vertical coordinates are signals. **Figure S13.** (A) The ceRNA regulatory network of EMT related genes. Rectangles, ellipses and hexagons indicate lncRNA, mRNA and miRNA, respectively. lncRNA and mRNA in EMT show significant up-regulation in red and down-regulation in blue. (B-D) Expression variation of GALNT1, USP53 and RHOBTB3 with pseudo-time. **Table S1.** COX regression (continuous) of EGR1 in other ovarian cancer datasets. **Table S2.** The samples information of GSE118828. **Table S3.** The samples information of GSE147082.**Table S4.** The samples information of GSE26712. **Table S5.** The samples information of TCGA-OV. **Table S6.** The basic information of tissue origin for SKOV3 and A2780 cell lines

## Data Availability

All the data used in the analysis can be obtained from TCGA, ENCODE, and GEO.
