# Supplementary material for: Characterization of tumour microenvironment reprogramming reveals invasion in epithelial ovarian carcinoma
Source: J Ovarian Res. 2023 Oct 10;16:200. doi: 10.1186/s13048-023-01270-7 (PMC10563280; doi:10.1186/s13048-023-01270-7)
Supplement: Supplementary file 1 — Additional file 1: Figure S1. (A) The histogram displays the distribution of counts detected per cell. The subplot in the upper right corner shows the distribution of the fraction of counts less than 3,000. Since here is a peak at count 700 and there are very few cells with counts greater than 20,000, the threshold is set to 500 and 15000. (B) The histogram displays the distribution of the number of genes detected per cell. There is a small peak at 300 genes (noise peak) and the threshold is set to 200. (C) The counts detected for each cell are plotted from high to low on the rank plot. 500 is the screening threshold since there is a rapidly decreasing inflection point at a counts count of 500. Figure S2. EOC cell distribution based on UMAP cluster plot. (A) The distribution of tumor and normal tissue cells. Each cell is labeled according to its origin. (B) The distribution of tumor cell states. (C) Copy number variations (CNVs) for per cell evaluated by InferCNV. Two normal-sample clusters and four stromal cell clusters were used as control group. Figure S3. Metabolic pathways and hallmark activity scores for cell subsets. (A) Metabolic pathway activity in each cell types. Values with low to high and statistically insignificant pathway activity (random permutation test p < 0.01) are shown as blank. (B) Hallmark activity in each cell types. Figure S4. The pathway and cancer hallmark activity characterization of different cell clusters. The P-values were calculated using one-way ANOVA. (A) The Glycolysis/Gluconeogenesis pathway activity status in different cell types. (B) The TCA cycle pathway activity status in different cell types. (C) The Oxidative phosphorylation pathway activity status in different cell types. (D) The HALLMARK_HYPOXIA pathway activity status in different cell types. Figure S5. Distribution of metabolic pathway activity scores in three clusters of epithelial cells. The difference in metabolic activity between the two is evaluated by Student's t t [file 13048_2023_1270_MOESM1_ESM.docx]

**Supplementary Figures**

**
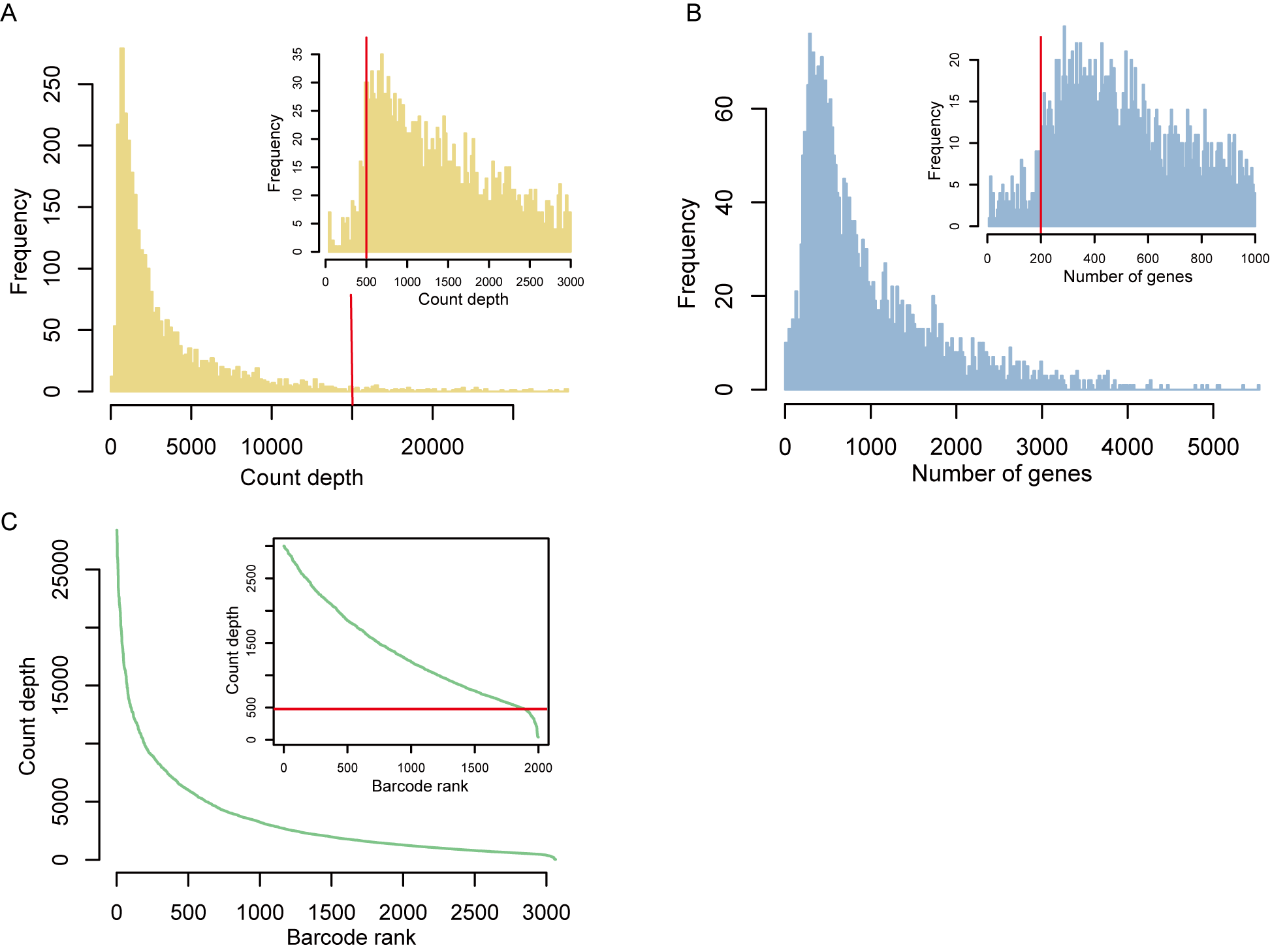
**

**Figure S1.** (A) The histogram displays the distribution of counts detected per cell. The subplot in the upper right corner shows the distribution of the fraction of counts less than 3,000. Since here is a peak at count 700 and there are very few cells with counts greater than 20,000, the threshold is set to 500 and 15000. (B) The histogram displays the distribution of the number of genes detected per cell. There is a small peak at 300 genes (noise peak) and the threshold is set to 200. (C) The counts detected for each cell are plotted from high to low on the rank plot. 500 is the screening threshold since there is a rapidly decreasing inflection point at a counts count of 500.


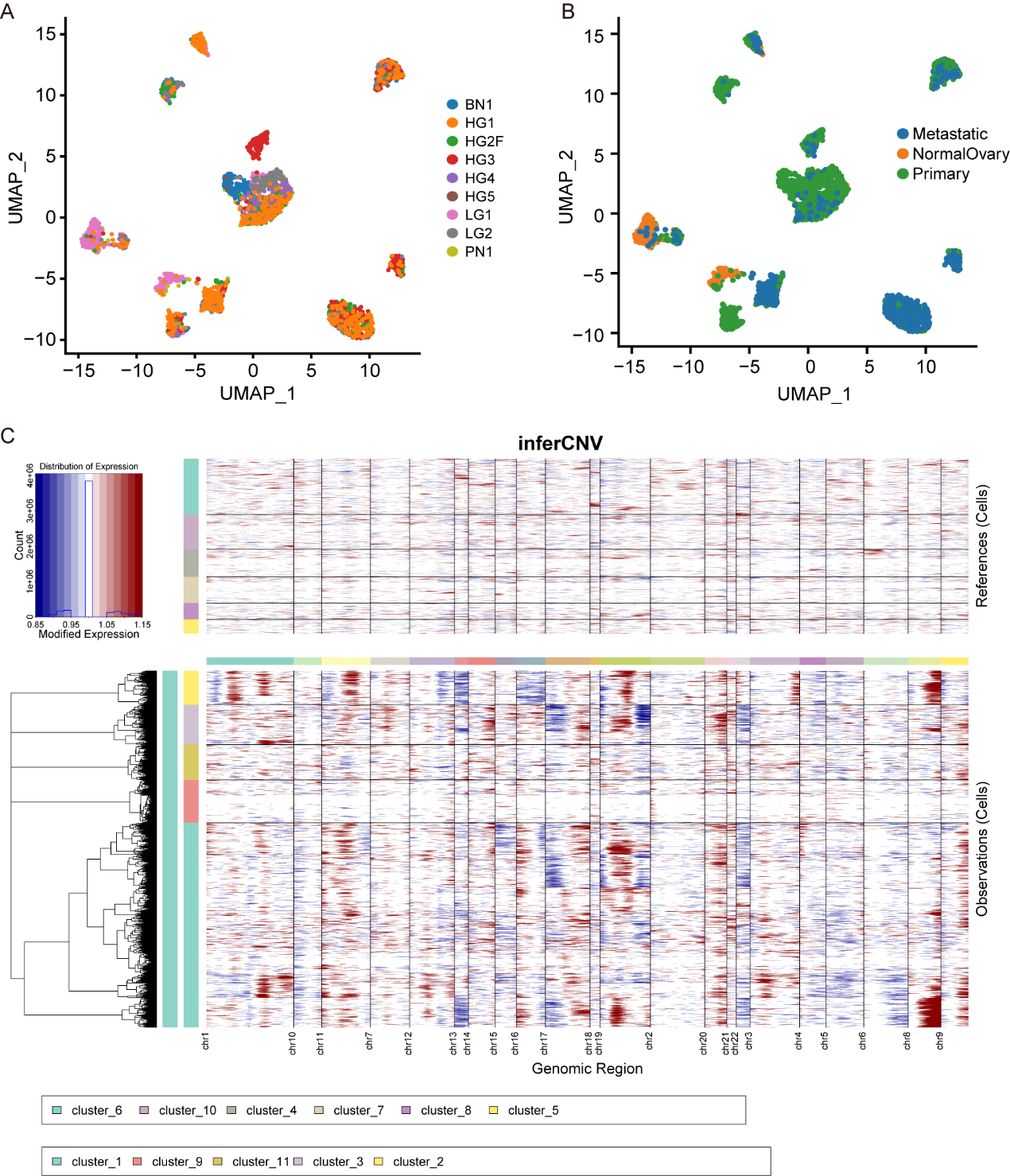


**Figure S2.** EOC cell distribution based on UMAP cluster plot. (A) The distribution of tumor and normal tissue cells. Each cell is labeled according to its origin. (B) The distribution of tumor cell states. (C) Copy number variations (CNVs) for per cell evaluated by InferCNV. Two normal-sample clusters and four stromal cell clusters were

used as control group.


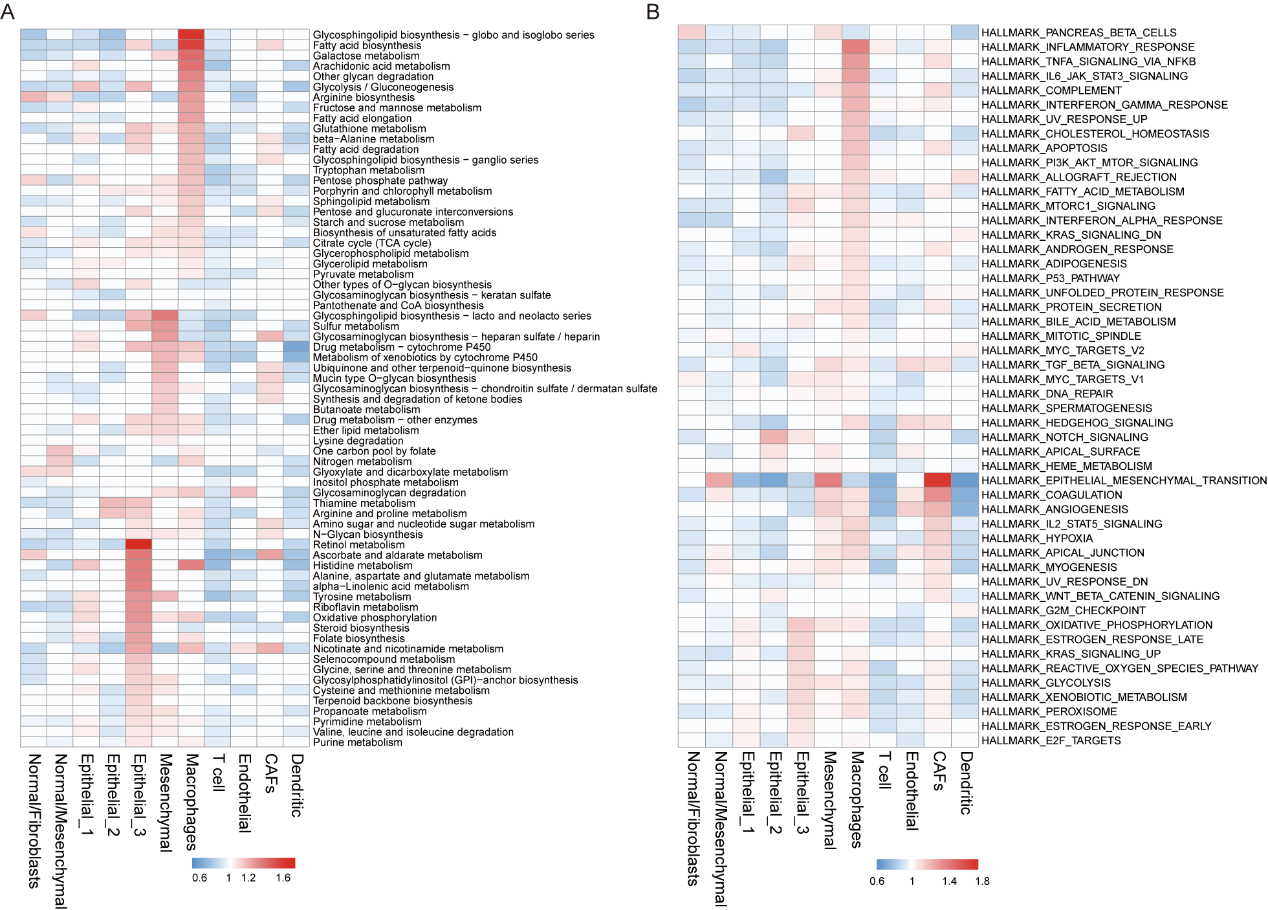


**Figure S3.** Metabolic pathways and hallmark activity scores for cell subsets. (A) Metabolic pathway activity in each cell types. Values with low to high and statistically insignificant pathway activity (random permutation test p < 0.01) are shown as blank. (B) Hallmark activity in each cell types.


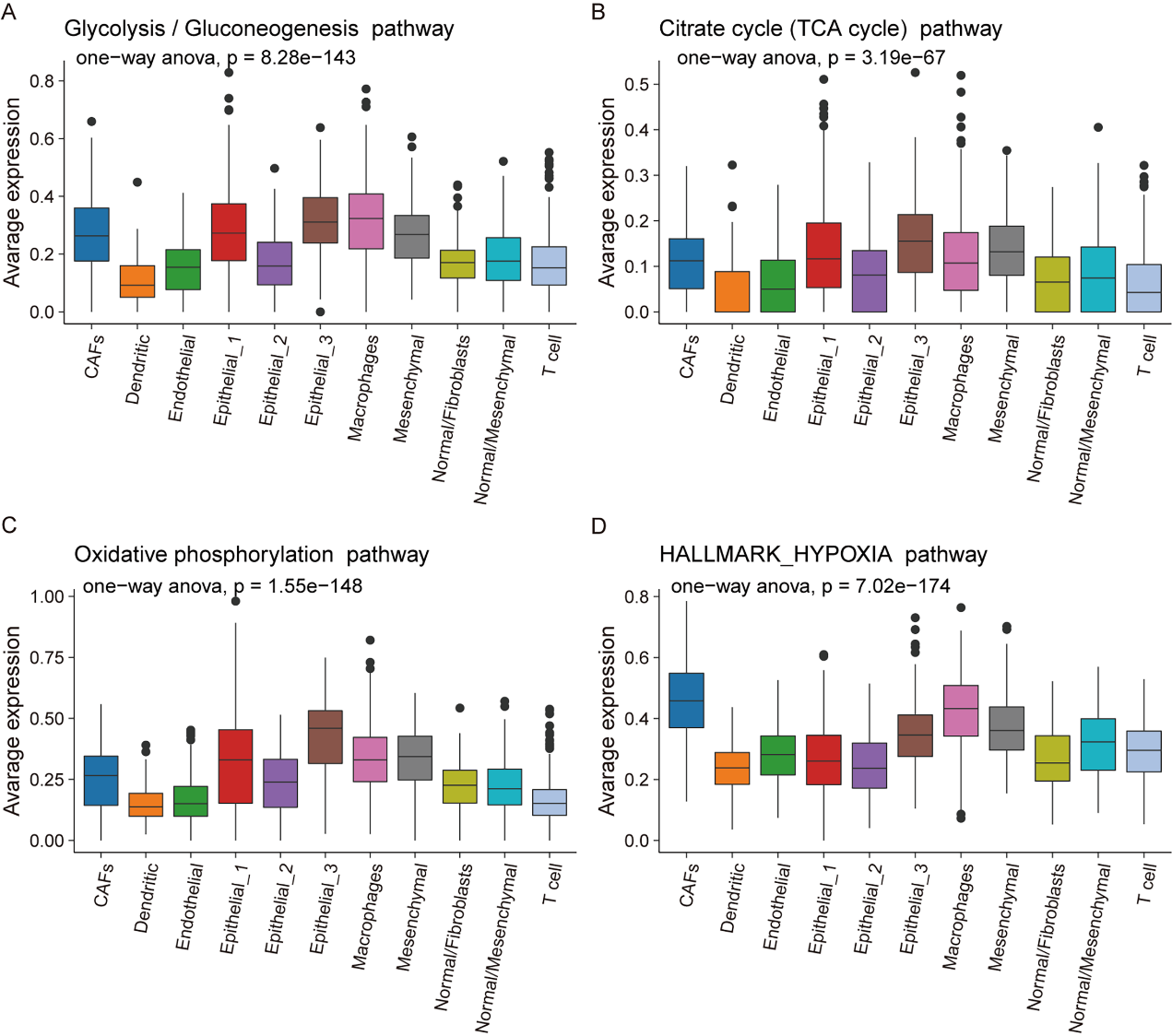


**Figure S4.** The pathway and cancer hallmark activity characterization of different cell clusters. The P-values were calculated using one-way ANOVA. (A) The Glycolysis/Gluconeogenesis pathway activity status in different cell types. (B) The TCA cycle pathway activity status in different cell types. (C) The Oxidative phosphorylation pathway activity status in different cell types. (D) The HALLMARK_HYPOXIA pathway activity status in different cell types.


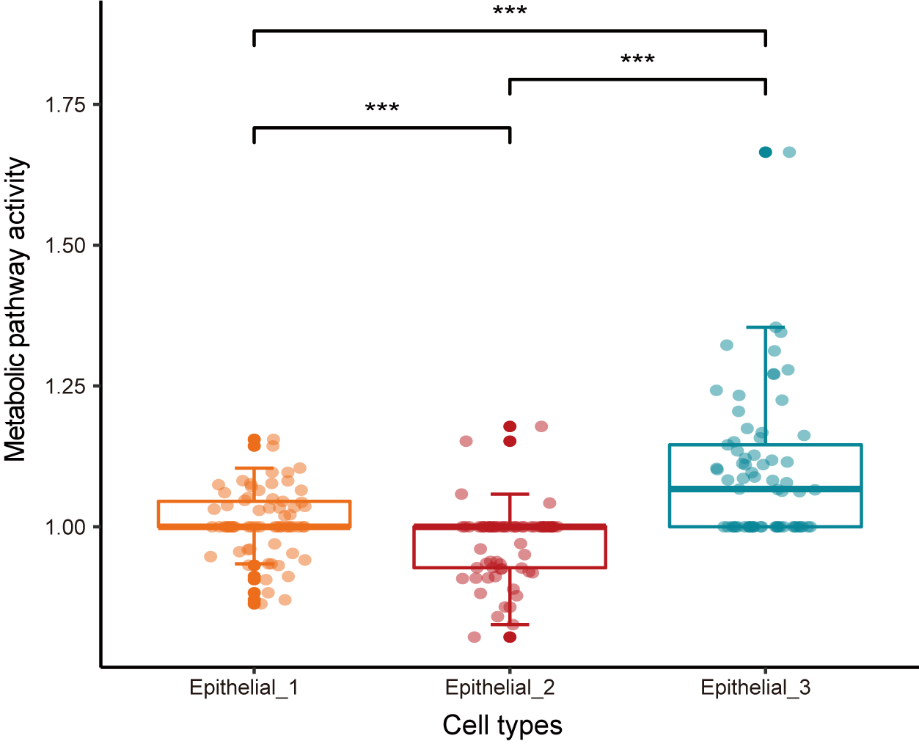


**Figure S5.** Distribution of metabolic pathway activity scores in three clusters of epithelial cells. The difference in metabolic activity between the two is evaluated by Student's t test.


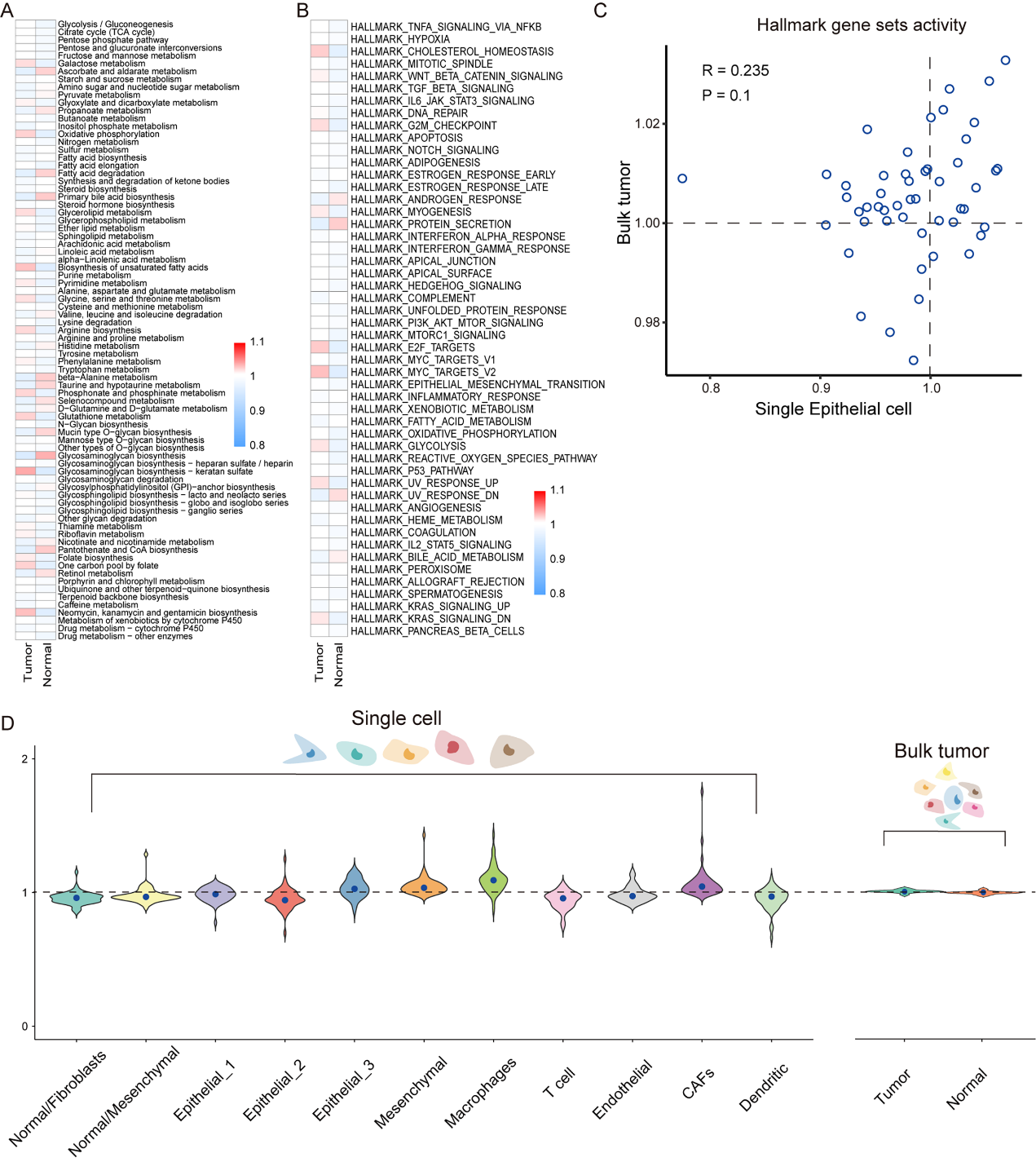


**Figure S6.** (A) Metabolic pathway activity in tumor and normal samples of bulk RNA-seq. White represents insignificant pathway activity scores (random permutation test p > 0.01). (B) The same as in A but for hallmark gene sets. (C) Scatter plot comparing hallmark gene sets activity between OV bulk tumors in TCGA and individual malignant cells in the scRNA-seq dataset. (D) The left panel shows the distribution of hallmark gene sets activity in different cell types in single-cell RNA-seq, and the right panel shows the hallmark gene sets activity in bulk tumor and normal samples from TCGA.

**
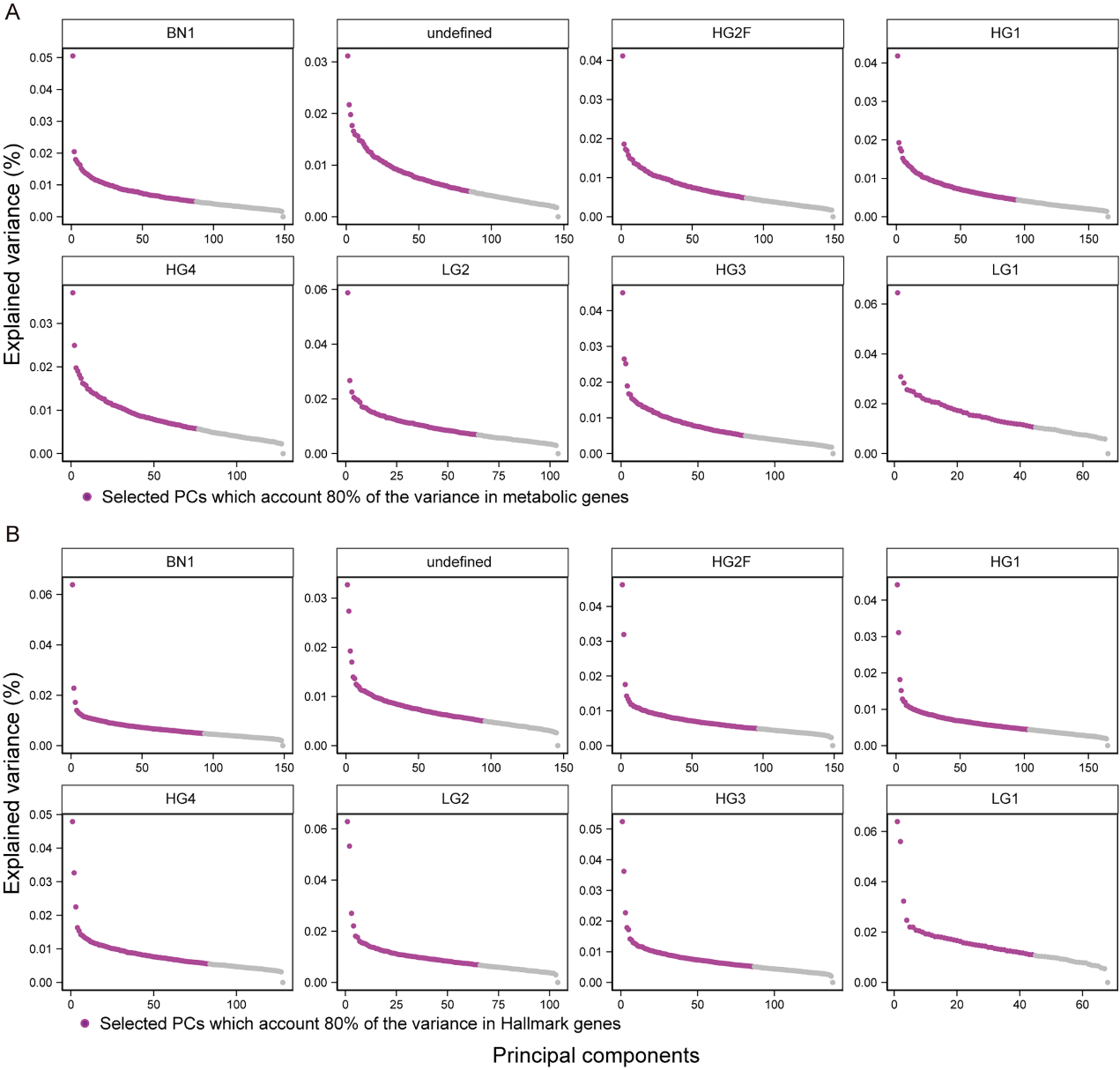
**

**Figure S7.** (A) Explanation of variance of principal components (PC) from principal component analysis (PCA) of metabolic gene expression levels in eight malignant epithelial cell clusters. Top PCs accounting for 80% of the variance are highlighted in purple. (B) The same as in A but for hallmark gene expression levels.

**
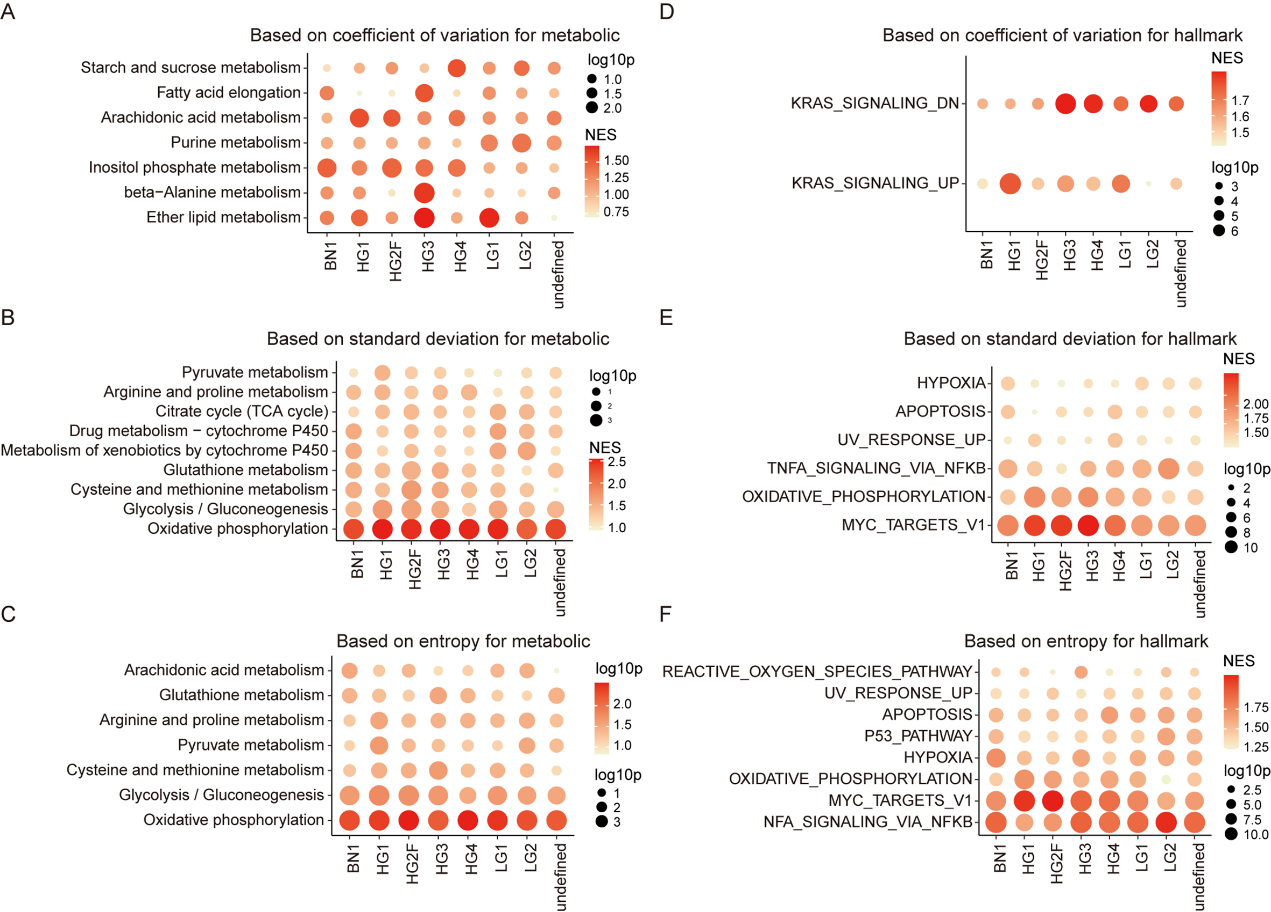
**

**Figure S8.** The scatter plot showing GSEA enrichment results for metabolic pathways and hallmarks. (A-C) The metabolic pathways were weighted by coefficient of variation, standard deviation and entropy, respectively. (D-E) The hallmarks were weighted by coefficient of variation, standard deviation and entropy, respectively.


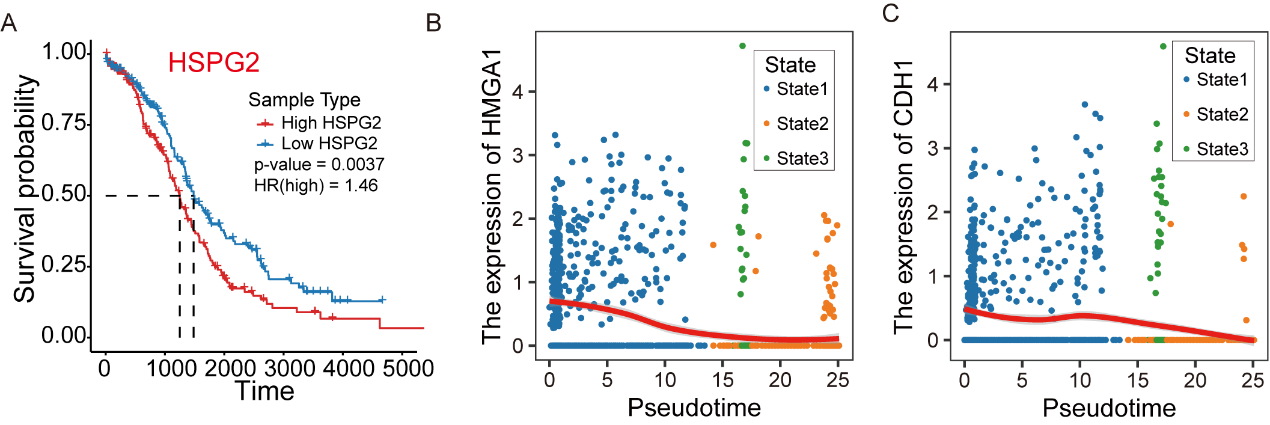


**Figure S9.** (A) Survival curves comparing high and low HSPG2 gene expression with patient overall survival (OS). (B-C) Variation of HMGA1 and CDH1, which are transcriptional regulatory pairs, expression levels with pseudo-time.


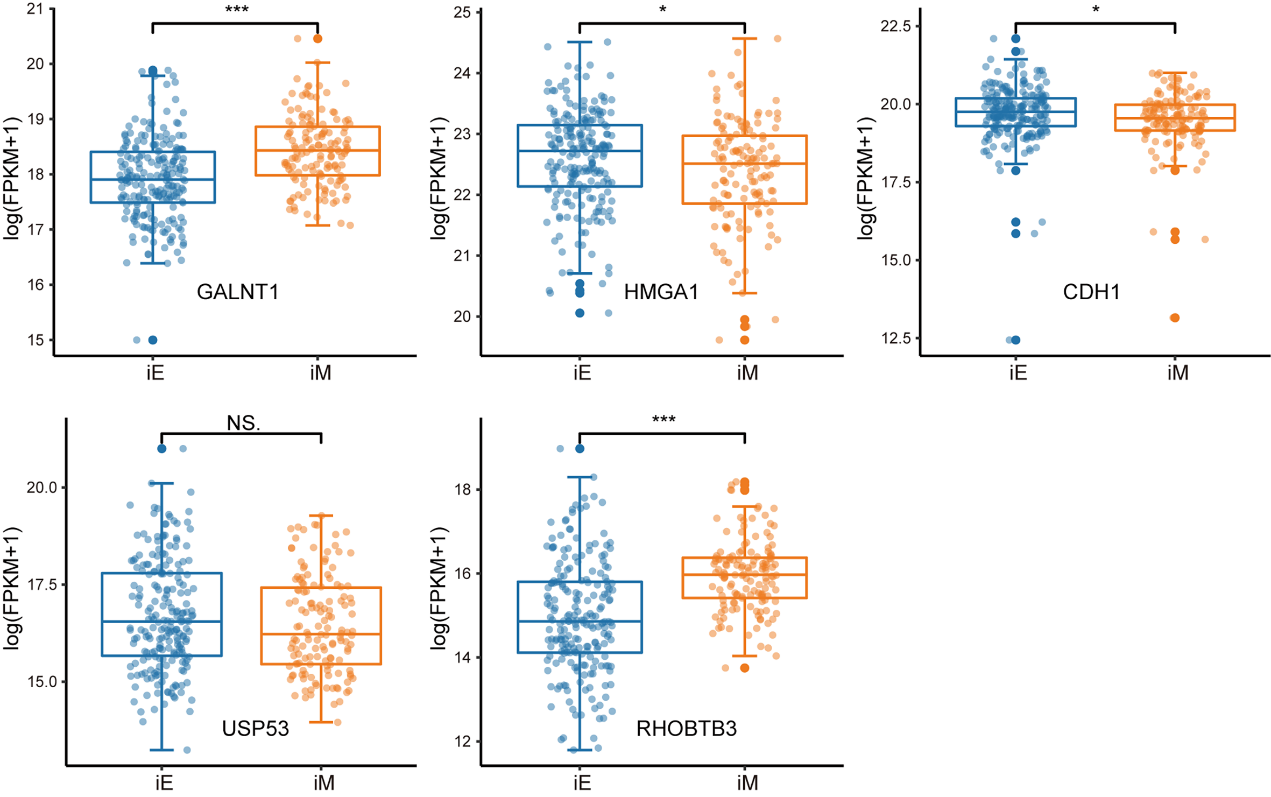


**Figure S10.** Box plot of genes including GALNT1, HMGA1, CDH1, USP53, and RHOBTB3 expression variations between invasive epithelial (iE) and invasive mesenchymal (iM) samples of bulk RNA-seq from TCGA.


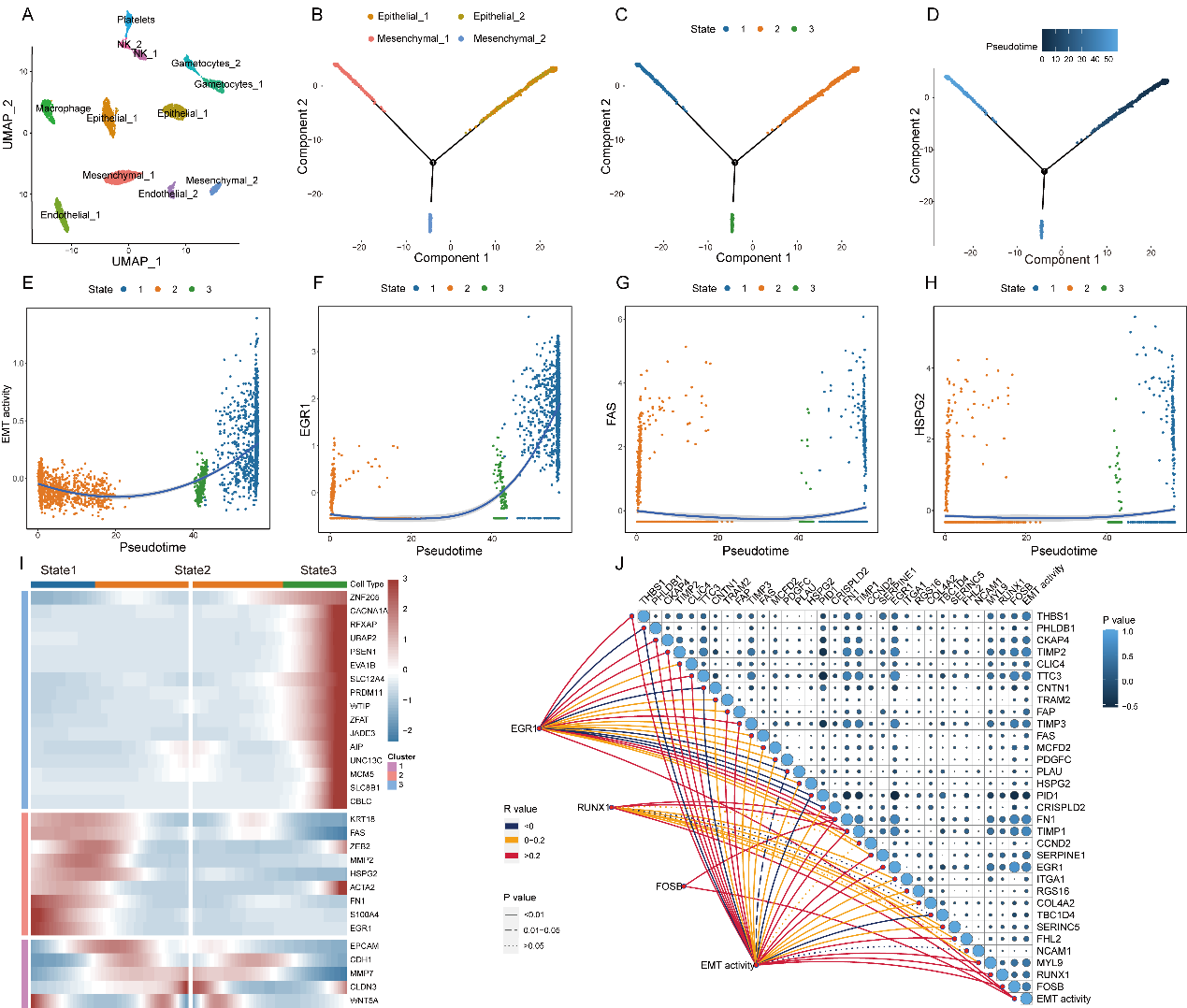


**Figure S11.** Validation of EMT process using an independent single-cell data (GSE147082). (A) The EOC cells were grouped into 13 clusters contain different cell types. (B-D) The epithelial cells and mesenchymal cells were mainly concentrated in separate branches and cellular states with continuously increased pseudo-time. (E-H) Increased EMT pathway activity, ERG1, FAS, and HSPG2 was found from state 2 to state 1 and 3. (F) Heatmap shows the branch-dependent genes at branch point 1. (G) Correlation heatmap between TF-target relationships and EMT activity.

**
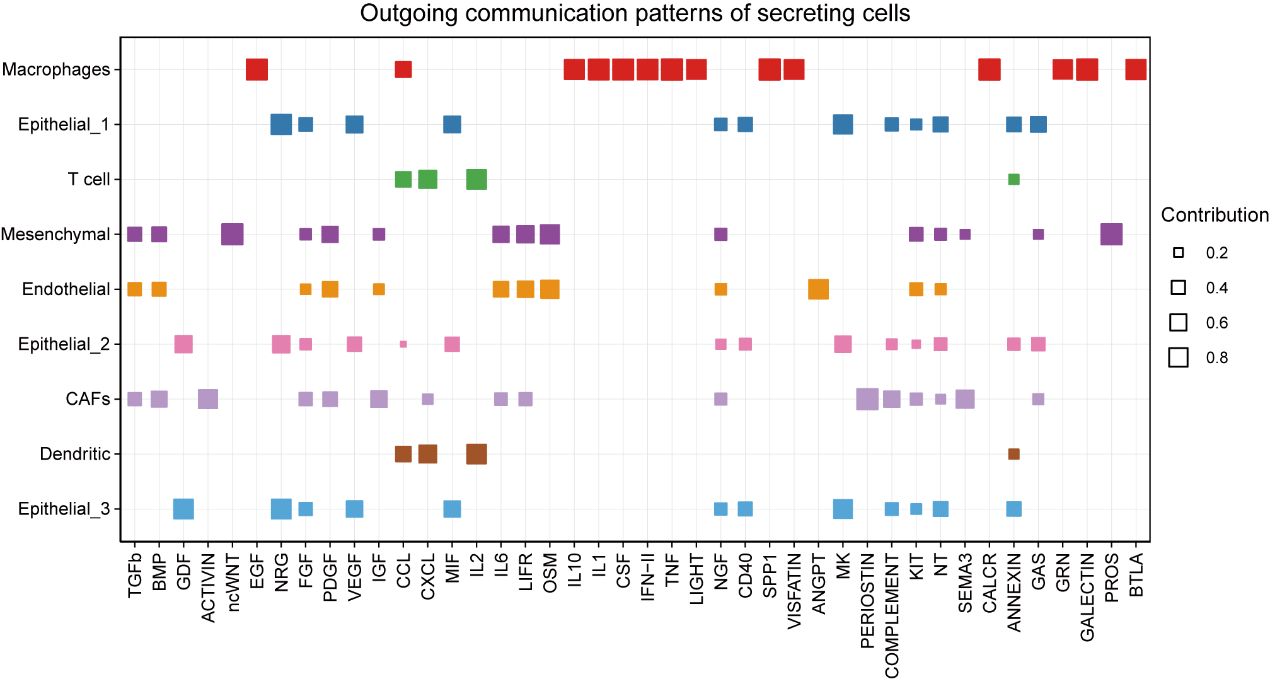
**

**Figure S12.** Contribution of nine cell types in ovarian cancer tissue communication. Horizontal coordinates are cell types and vertical coordinates are signals.


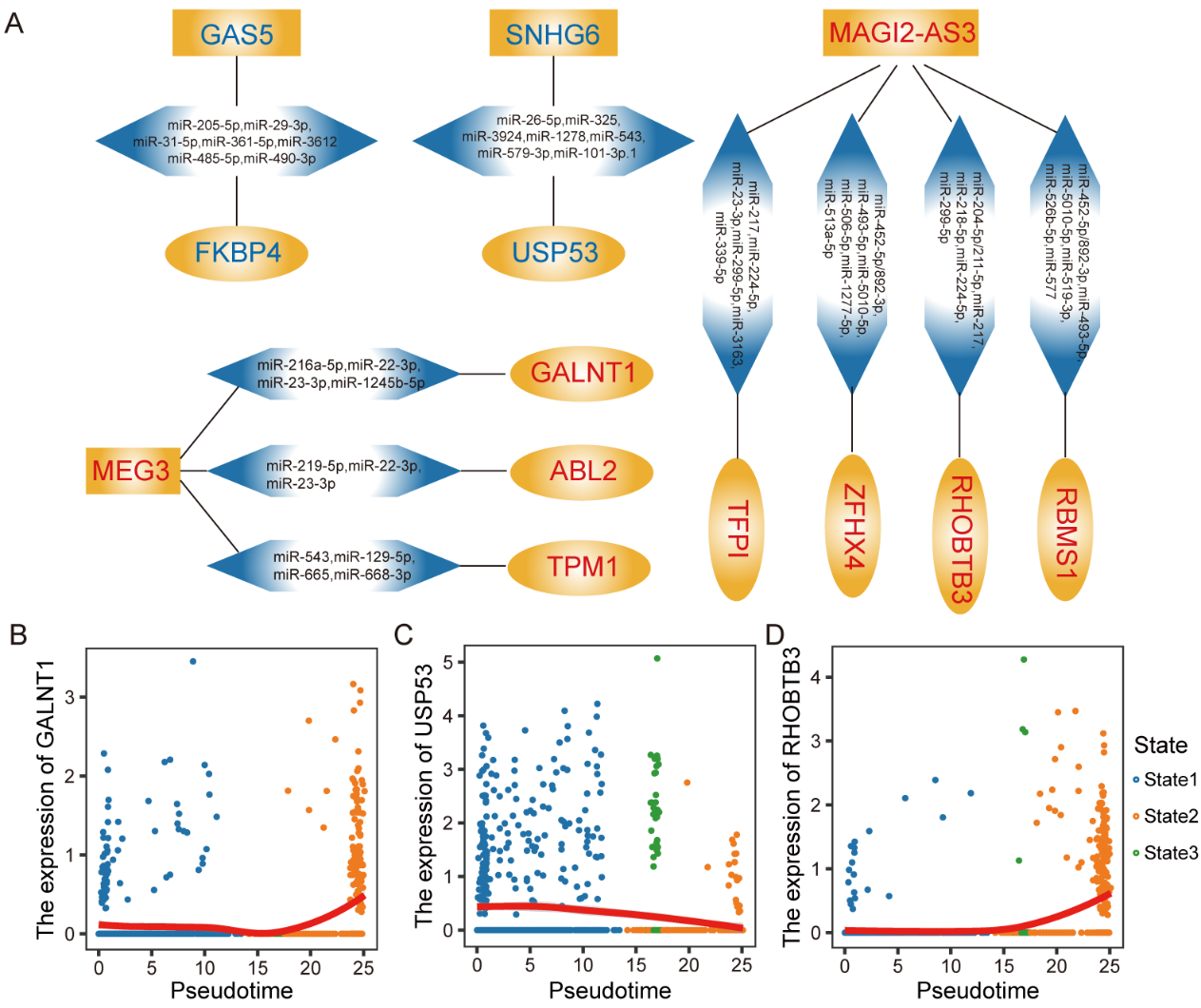


**Figure S13.** (A) The ceRNA regulatory network of EMT related genes. Rectangles, ellipses and hexagons indicate lncRNA, mRNA and miRNA, respectively. lncRNA and mRNA in EMT show significant up-regulation in red and down-regulation in blue. (B-D) Expression variation of GALNT1, USP53 and RHOBTB3 with pseudo-time.

**Supplementary Tables**

**Table S1.** COX regression (continuous) of EGR1 in other ovarian cancer datasets

| Gene | Datasets | Treatment | Survival  Type | HR | 95%Cl | P-value |
| --- | --- | --- | --- | --- | --- | --- |
| EGR1 | GSE30161 | Chemotherapy | OS | 1.49 | 1.008-2.218 | 4.58e-02 |
|  |  |  | PFS | 1.41 | 1.170-1.705 | 3.30e-04 |
|  | GSE49997 | Chemotherapy | OS | 1.36 | 1.039-1.769 | 2.52e-02 |
|  | TCGA-OV | Chemo /Immune/Hormone | PFI | 1.11 | 1.007-1.223 | 3.61e-02 |
|  | GSE26193 | Chemotherapy | PFS | 1.21 | 1.005-1.451 | 4.42e-02 |
|  | GSE51088 | Chemotherapy | OS | 1.49 | 1.008-2.218 | 4.58e-02 |
|  | GSE8842 | Surgery/Chemo | OS | 1.22 | 0.983-151.49 | 5.15e-02 |

**Table S2**. The samples information of GSE118828

| Patient | Sample | GEO id | Tumor type | Origin |
| --- | --- | --- | --- | --- |
| P1 | PN1-P | GSM3348303 | Peritonal | Primary |
| P2 | BN1-P | GSM3348304 | Benign | Primary |
| P3 | NA1-P | GSM3348306 | HGSOC | Primary |
| P4 | NM1 | GSM3348307 | Normal | Normal Ovary |
|  | LG1-P | GSM3348308 | LGSOC | Primary |
| P5 | HG2F-P | GSM3348309 | HGSOC | Primary |
|  | HG2F-M | GSM3348310 | HGSOC | Metastatic |
| P6 | HG3-M1 | GSM3348305 | HGSOC | Metastatic |
|  | HG3-P | GSM3348311 | HGSOC | Primary |
|  | HG3-M2 | GSM3348312 | HGSOC | Metastatic |
| P7 | HG4-P1 | GSM3348313 | HGSOC | Primary |
|  | HG4-P2 | GSM3348314 | HGSOC | Primary |
|  | HG4-P3 | GSM3348315 | HGSOC | Primary |
|  | HG4-P4 | GSM3348316 | HGSOC | Primary |
| P8 | LG2-M | GSM3348317 | LGSOC | Metastatic |
|  | LG2-P | GSM3348318 | LGSOC | Primary |
| P9 | HG1-P | GSM3348319 | HGSOC | Primary |
|  | HG1-M | GSM3348320 | HGSOC | Metastatic |

**Table S3.** The samples information of GSE147082

| Patients | GEO id | Origin | Tumor type | neoadjuvant therapy | Age |
| --- | --- | --- | --- | --- | --- |
| P1 | GSM4416534 | Left fallopian (STIC) | HGSOC | No | 46 |
| P2 | GSM4416535 | undetermined | Serous | Yes | 62 |
| P3 | GSM4416536 | Left fallopian (STIC) | HGSOC | Yes | 66 |
| P4 | GSM4416537 | Left fallopian (STIC) | HGSOC | Yes | 71 |
| P5 | GSM4416538 | Left ovary | HGSOC | No | 56 |
| P6 | GSM4416539 | Fallopian | Mixed | Yes | 66 |

**Table S4**. The samples information of GSE26712

|  | Subtype | Number | Ratio |
| --- | --- | --- | --- |
| Sample Type | Normal | 10 | 5.13% |
|  | Tumor | 185 | 94.87% |
| Tumor type | Normal | 10 | 5.13% |
|  | HGSOC | 185 | 94.87% |
| Survival Time | < 2 Year | 64 | 32.82% |
|  | >2 Year and < 5 Year | 85 | 43.59% |
|  | > 5 Year | 36 | 18.46% |

**Table S5.** The samples information of TCGA-OV

|  | Subtype | Number | Ratio |
| --- | --- | --- | --- |
| Stage | II | 21 | 5.63% |
|  | III | 292 | 78.28% |
|  | IV | 57 | 15.28% |
|  | Undefined | 3 | 0.80% |
| Invasion | Yes | 62 | 16.62% |
|  | No | 40 | 10.72% |
|  | Undefined | 271 | 72.65% |
| Residual Disease | No disease | 64 | 17.16% |
|  | 1-10 mm | 171 | 45.84% |
|  | 11-20 mm | 26 | 6.97% |
|  | >20 mm | 70 | 18.77% |
|  | Undefined | 42 | 11.26% |
| Survival Status | 0 | 143 | 38.34% |
|  | 1 | 230 | 61.66% |
| Survival Time | < 2 Year | 139 | 37.27% |
|  | >2 Year and < 5 Year | 161 | 43.16% |
|  | > 5 Year | 73 | 19.57% |

**Table S6**. The basic information of tissue origin for SKOV3 and A2780 cell lines

| Cell lines | Organism | Tissue | Age | Morphology | Growth properties | Disease |
| --- | --- | --- | --- | --- | --- | --- |
| SKOV3 | Homo sapiens | Ovary; Ascites | 64 | epithelial | Adherent | Serous cystadenocarcinoma |
| A2780 | Homo sapiens | Ovary | / | epithelial | Adherent | endometroid  adenocarcinoma |
